# Current and future immunotherapeutic approaches in pancreatic cancer treatment

**DOI:** 10.1186/s13045-024-01561-6

**Published:** 2024-06-04

**Authors:** Pooya Farhangnia, Hossein Khorramdelazad, Hamid Nickho, Ali-Akbar Delbandi

**Affiliations:** 1 Reproductive Sciences and Technology Research Center, Department of Immunology, School of Medicine, Iran University of Medical Sciences, Tehran, Iran; 2 Immunology Research Center, Institute of Immunology and Infectious Diseases, Iran University of Medical Sciences, Tehran, Iran; 3 Department of Immunology, School of Medicine, Iran University of Medical Sciences, Tehran, Iran; 4 Immunology Board for Transplantation and Cell-Based Therapeutics (ImmunoTACT), Universal Scientific Education and Research Network (USERN), Tehran, Iran; 5 Department of Immunology, School of Medicine, Rafsanjan University of Medical Sciences, Rafsanjan, Iran

**Keywords:** Pancreatic cancer immunotherapy, Pancreatic ductal adenocarcinoma, Adoptive cell therapy, CAR NK cell therapy, CAR T-cell therapy, Immune checkpoint blockade, Immune checkpoint inhibitor, Oncolytic virus therapy, Cancer vaccine

## Abstract

Pancreatic cancer is a major cause of cancer-related death, but despondently, the outlook and prognosis for this resistant type of tumor have remained grim for a long time. Currently, it is extremely challenging to prevent or detect it early enough for effective treatment because patients rarely exhibit symptoms and there are no reliable indicators for detection. Most patients have advanced or spreading cancer that is difficult to treat, and treatments like chemotherapy and radiotherapy can only slightly prolong their life by a few months. Immunotherapy has revolutionized the treatment of pancreatic cancer, yet its effectiveness is limited by the tumor's immunosuppressive and hard-to-reach microenvironment. First, this article explains the immunosuppressive microenvironment of pancreatic cancer and highlights a wide range of immunotherapy options, including therapies involving oncolytic viruses, modified T cells (T-cell receptor [TCR]-engineered and chimeric antigen receptor [CAR] T-cell therapy), CAR natural killer cell therapy, cytokine-induced killer cells, immune checkpoint inhibitors, immunomodulators, cancer vaccines, and strategies targeting myeloid cells in the context of contemporary knowledge and future trends. Lastly, it discusses the main challenges ahead of pancreatic cancer immunotherapy.

## Introduction

Pancreatic cancer comprises mostly pancreatic ductal adenocarcinoma (PDAC), a persistent and recalcitrant disease [1], and is responsible for an estimated 50,550 deaths in the United States of America in 2023 [2]. Diagnosis in the early stages of metastasis or late-stage is common since symptoms are often vague. The current approach for treating PDAC is standard cytotoxic chemotherapy, but it only extends overall survival (OS) by a few months [3–5].

PDAC carcinogenesis like all the solid tumors is mediated by the gradual build-up of driver mutations, such as the oncogene KRAS (G12D mutation) [6–9] and the tumor suppressor gene TP53 [10, 11]. These molecular modifications are accompanied by corresponding histological alterations during different stages of PDAC development [12]. The morphological progression initiates with the formation of precursor lesions known as pancreatic intraepithelial neoplasia (PanIN) [13], which then advance to invasive adenocarcinoma. Changes in the surrounding tissue stroma occur as cancer continues to advance. The non-transformed tissue stroma, composed of components such as immunological, vascular, and connective tissue, plays a vital role in maintaining homeostasis in response to damage. However, cancer exploits these physiological responses to create a favorable tumor microenvironment (TME) for its efficient growth [12, 14]. Indeed, cancer resembles "persistent wounds", and alterations in the stroma are the outcome of "abnormal wound healing" [15].

Immunotherapeutic strategies possess a significant capability in inducing strong immune responses against tumors. Immunomodulators, immune checkpoint blockade (ICB), and adoptive cell transfer therapy could potentially offer hopeful strategies [16–18]. Remarkable outcomes have been achieved from 2010 to the present through clinical research that utilizes various immunotherapeutic approaches to treat patients with different types of cancer [19–22]. The immune responses specifically targeting cancer cells, triggered by immunotherapy, differ from those stimulated by tumor-directed therapies. Furthermore, these responses can endure for a prolonged period even after the treatment is discontinued [23, 24]. However, the application of immunotherapy yields insufficient results for the vast majority of PDACs. This is predominantly attributed to the characteristics of its TME, which is deficient in effector T cells that have previously been exposed to antigens [25].

Tumor immunotherapy has revolutionized the treatment of various solid tumors. Nevertheless, current immunotherapies have had limited success in improving survival for patients with PDAC [26, 27]. The immunological resistance of PDAC to immunotherapies can be attributed to its low mutational burden and the hostile TME characterized by fibrosis, hypoxia, and immunosuppression [28–30]. However, a meta-analysis suggested that targeted immunotherapy is more effective than standard treatments in increasing survival and enhancing immune responses in pancreatic cancer patients [31]. Moreover, combining chemotherapy and surgery with other immunotherapies may synergistically improve outcomes. Various cytotoxic drugs and adjuvant therapies have been shown to sensitize the TME to immunotherapy by inducing immunogenic cell death, modifying evasive immune processes, and reducing immune suppression [32, 33].

Immunotherapy is presently emerging as a focal point in the treatment of pancreatic cancer. This persistent tumor primarily escapes immune detection through various means, including the secretion of immunosuppressive factors like transforming growth factor-beta (TGF-β), the creation of an immunosuppressive environment lacking T lymphocytes, and the expression of immune checkpoints such as programmed death-ligand 1 (PD-L1) and PD-L2 [4, 34]. Furthermore, research is being conducted on ICB to activate T-cell function in pancreatic cancer [35–37]. The pancreatic cancer microenvironment is characterized by extensive desmoplasia, a scarcity of effector T lymphocytes, and an immunophenotype dominated by T helper 2 (TH2) cells, all of which facilitate the evasion of cancer cells from immune surveillance [38–40]. Consequently, monoclonal antibodies (mAbs) targeting programmed cell death protein 1 (PD-1) and PD-L1 have shown limited efficacy [4]. Moreover, immunotherapies like PD-1 inhibition may benefit only a small percentage of cancer patients (3%) who have hyper-mutation and microsatellite instability [41].

This article delves headfirst into a comprehensive analysis of the immunosuppressive microenvironment in pancreatic cancer. In the context of contemporary knowledge and future trends, the article elaborates on a wide range of immunotherapies, such as oncolytic virus therapy (OVT), adoptive cell transfer therapy including T-cell receptor (TCR)-engineered T cells therapy, chimeric antigen receptor (CAR) T-cell therapy, CAR natural killer (NK) cell therapy, and cytokine-induced killer cells. Additionally, it examines immune checkpoint inhibitors (ICIs) and immunomodulators, cancer vaccines, and immunotherapeutic approaches that target myeloid cells. Lastly, the article highlights the effects of the gut microbiome in modulating response to ICIs and the emerging role of CRISPR/Cas9 gene-editing technology in pancreatic cancer immunotherapy. Finally, it discusses the main challenges ahead of pancreatic cancer immunotherapy.

## Exploring the tumor microenvironment (TME) of pancreatic cancer

**Fig. 1:**
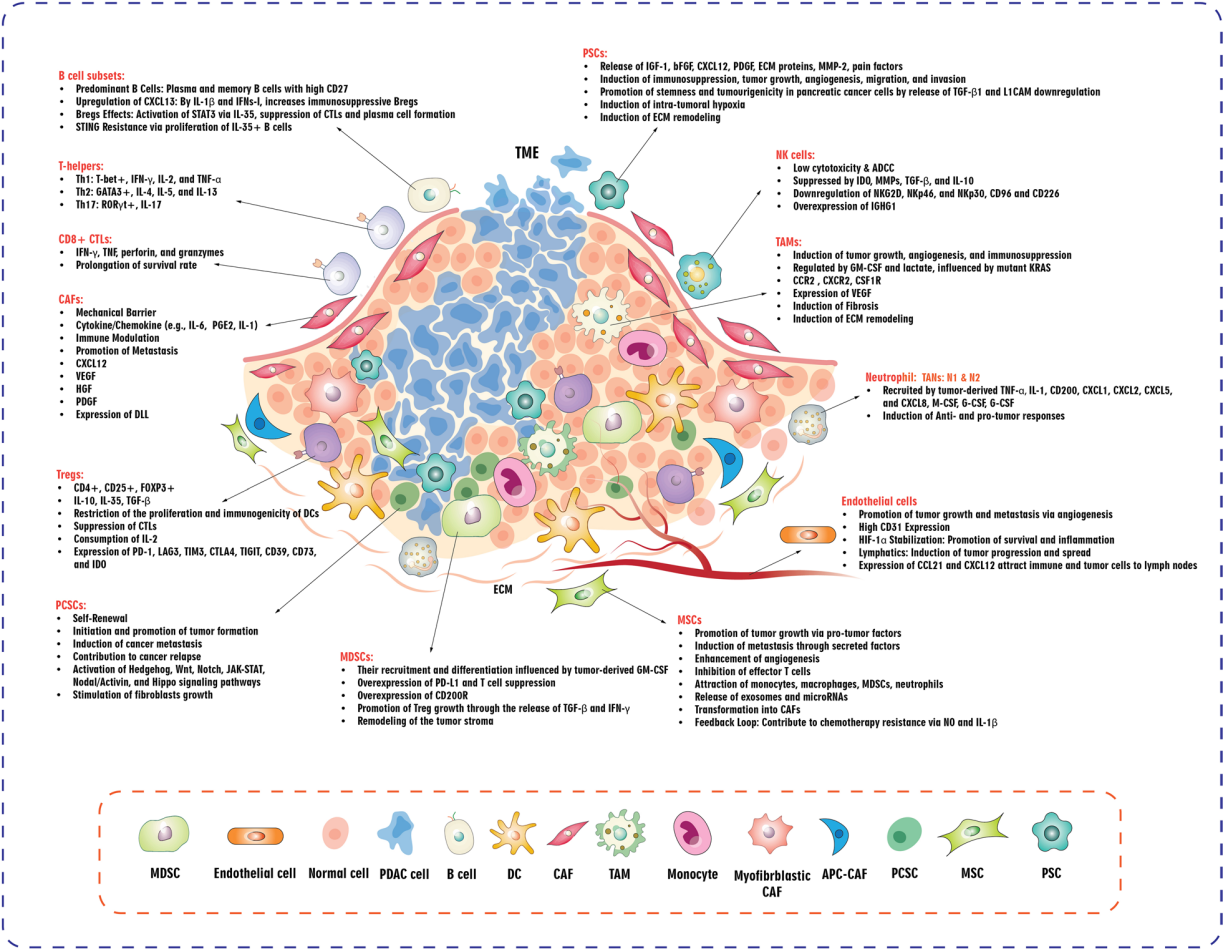
Tumor microenvironment (TME) in pancreatic cancer. ADCC: Antibody-dependent cellular cytotoxicity; APC: Antigen-presenting cell; CAF: Cancer-associated fibroblast; CTL: Cytotoxic T lymphocyte; DC: Dendritic cell; DLL: Delta like canonical notch ligand; ECM: Extracellular matrix; GM-CSF: Granulocyte–macrophage colony-stimulating factor; HGF: Hepatocyte growth factor; IDO: Indoleamine 2,3-dioxygenase; IFNs-I: Type I interferons; IFN-γ: Interferon-gamma; IL-2: Interleukin 2; MDSC: Myeloid-derived suppressor cell; MMP: Matrix metalloproteinase; MQ: Macrophage; MSC: Mesenchymal stromal cell; NK: Natural killer; NO: Nitric oxide; PCSC: Pancreatic cancer stem cell; PDAC: Pancreatic ductal adenocarcinoma; PDGF: Platelet-derived growth factor; PSC: Pancreatic stellate cell; STING: Stimulator of interferon genes; TAM: Tumor-associated macrophage; TAN: Tumor-associated neutrophil; TGF-β: Transforming growth factor beta; Th1: Type 1 T helper; TNF-α: Tumor necrosis factor alpha; Treg: Regulatory T cell; VEGF: Vascular endothelial growth factor

**Fig. 2:**
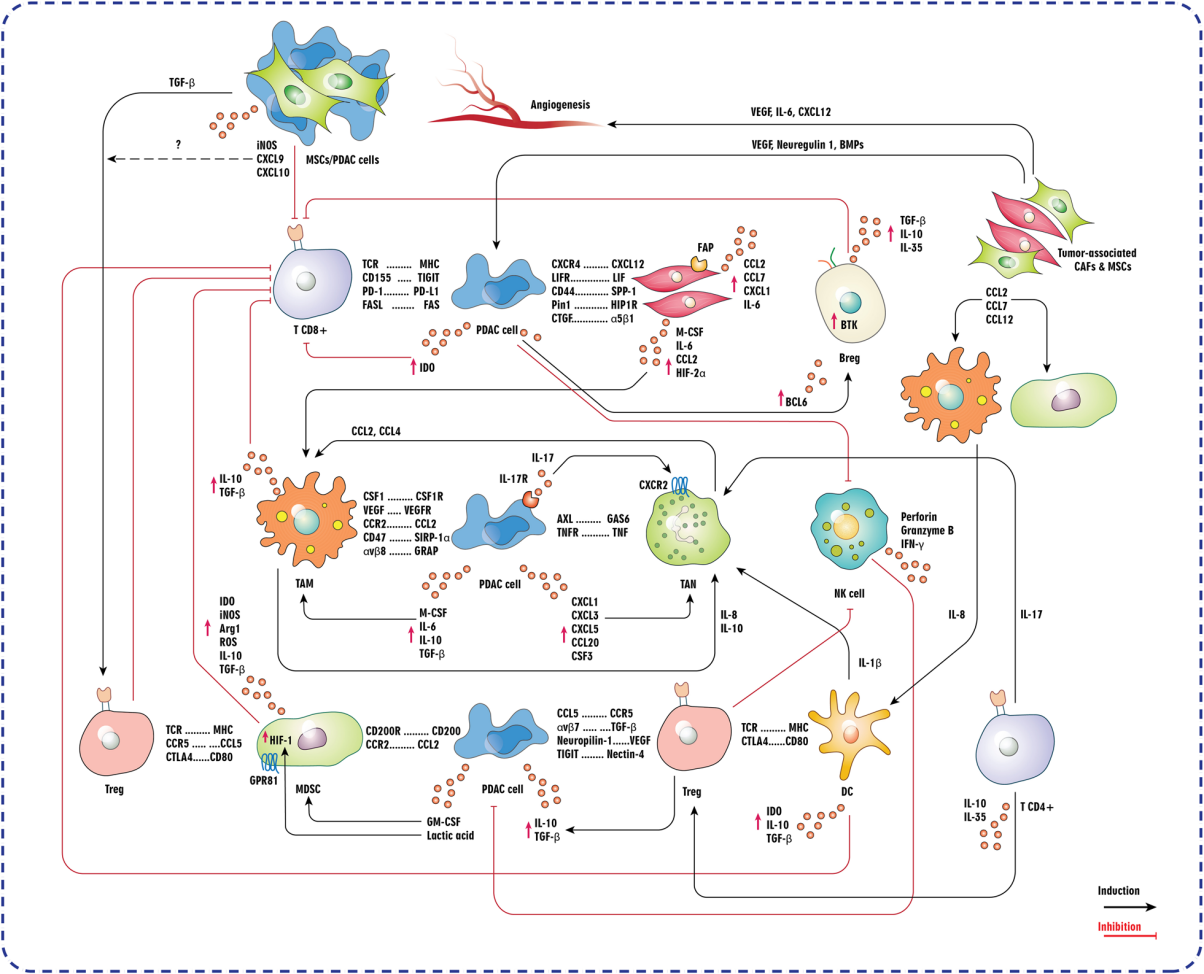
Crosstalk between pancreatic ductal adenocarcinoma (PDAC) cells and key components of tumor microenvironment (TME). Arg1: Arginase 1; BMPs: Bone morphogenetic proteins; Breg: Regulatory B cell; BTK: Bruton's tyrosine kinase; CAFs: Cancer-associated fibroblast; CSF1: Colony stimulating factor 1; CTGF: Connective tissue growth factor; DC: Dendritic cell; FAP: Fibroblast activation protein; HIF: Hypoxia-inducible factor; IDO: Indoleamine 2,3-dioxygenase; iNOS: Inducible nitric oxide synthase; LIF: Leukemia inhibitory factor; M-CSF: Macrophage colony-stimulating factor; MDSC: Myeloid-derived suppressor cell; MHC: Major histocompatibility complex; MSCs: Mesenchymal stem/stromal cells; NK: Natural killer; Pin1: Peptidylpropyl isomerase; ROS: Reactive oxygen species; SPP-1: Osteopontin/secreted phosphoprotein 1; TAM: Tumor-associated macrophage; TAN: Tumor-associated neutrophil; TCR: T cell receptor; TGF-β: Transforming growth factor beta; TIGIT: T cell immunoreceptor with Ig and ITIM domains; TNF: Tumor necrosis factor; Treg: Regulatory T cell; VEGF: Vascular endothelial growth factor

The complicated interaction between tumor cells and their adjacent microenvironment significantly impacts the development of solid tumors. Determining the outcome of cancer, whether it progresses or regresses, heavily relies on the immune environment present in tumors. This environment is made up of various cell types such as adaptive immune cells, macrophages, dendritic cells (DCs), NK cells, and other innate immune cells [42]. PDAC serves as a prime example of the various types of communication that can occur between tumors and surrounding tissue. PDAC demonstrates strong resistance to new immunotherapies due to the exclusive collaboration between different immune cells, resulting in the creation of a highly immunosuppressive setting that aids tumor advancement [12, 43–46]. The "cold" TME is a distinct feature of a pancreatic tumor wherein a considerable infiltration of myeloid cells is observed, and CD8^+^ T cells are usually absent, resulting in immunological characteristics [47]. Given the heterogeneous nature of pancreatic TME, components may have dual, contradicting roles (Table 1). In this section, we outline the involvement of immune cells and non-immune cells in the TME of pancreatic cancer and cross-talk between these cells (Figs. 1 and 2).

**Table 1:** Dual role of key components of pancreatic tumor microenvironment

Component	Pro-tumor effects	Anti-tumor/limiting effects
T lymphocytes	CD4^+^ T cell supported cancer progression by secreting IL-17 and IL-27 [551, 552]	Cytotoxic TILs induced tumor regression [553, 554]
Regulatory T lymphocytes (Tregs)	Treg suppressed immunity against early stage pancreatic intraepithelial neoplasms [555]	Treg depletion led to accelerated tumor progression [104]
B lymphocytes	B cells supported tumor progression/proliferation by secreting IL-35 and activating immunosuppressive TAMs [556, 557]	Insufficient data
Myeloid cells	CD11b^+^ myeloid cells are required for oncogenic Kras-driven PanIN formation [165, 166, 558]	Reinvigorating dysregulated myeloid cells in therapeutic settings (e.g., using CD40 agonist) [130, 407, 431, 559]
CAF	Regulating tumor metabolism for cancer cell proliferation and suppressing anti-tumor immunity [35, 560–563]	Increased matrix deposition and forming a dense and stiff matrix around early PDAC cells [242, 564, 565]
ECM	Supporting cancer cell proliferation and migration [566, 567]	Cancer-cell-derived fibrillar collagen and type I collagen restrains tumor growth [568, 569]

CAF: Cancer-associated fibroblast, ECM: Extracellular matrix, PanIN: Pancreatic intraepithelial neoplasia, PDAC: Pancreatic ductal adenocarcinoma, TAMs: Tumor-associated macrophages, TILs: Tumor-infiltrating lymphocytes

### The role of immune cells

The TME comprises various immune cells, each with distinct roles and significance. This section will elucidate the functions of these immune cells within the TME.

#### Role of T lymphocytes in TME

The immunological diversity among tumors in patients with PDAC is wide-ranging, characterized by varying densities of infiltrating T-cells and the composition of T-cell subpopulations [48–51]. The presence of desmoplastic elements might not influence the accumulation of T cells, thus revealing a separate spatial arrangement of T cells in PDAC [50]. This challenges the idea that the inhibitory environment shaped by fibroblasts and desmoplastic stroma suppresses the infiltration of T cells [52, 53]. In pancreatic tumors, the extravasation of T cells is constrained by the desmoplastic stroma [54], leading to immune exclusion, the induction of immunosuppression, and the inefficacy of anti-cancer therapies [55].

The presence of more CD8^+^ cytotoxic T lymphocytes (CTLs) encircling cancerous cells is associated with a boost in the survival rates of patients [50]. According to the study, in patients who had a better survival, tumor samples exhibited a greater percentage of CD8^+^ T cells, but a lesser percentage of CD4^+^ T cells compared to tumor samples from patients with a short survival [51]. These results highlight the complexity of the immune response in PDAC and raise questions about the role of the TME in shaping immune profiles. Further investigation is needed to fully understand these findings and their implications for future treatments. In the subsequent discourse, we explicate the pivotal contribution of T cells in the TME according to distinct T cell phenotypes.

##### Cytotoxic T lymphocytes (CTLs)

The principal participants in the battle against cancer cells are the CTLs that produce IFN-γ, TNF, perforin, and granzymes. These CTLs are responsible for generating durable memory cells that grant protection against cancer cells in the times to come. CTLs can recognize and kill tumor cells that express cognate tumor antigens. This specific recognition is achieved through the interaction between the TCR on CTLs and the peptide-major histocompatibility complex (MHC) on the tumor cell surface. Once the recognition occurs, CTLs induce the death of the target cell through apoptosis [56].

Previous research has demonstrated that the prognosis of individuals diagnosed with pancreatic cancer is influenced by the distribution of CD8^+^ TILs [57]. Increased survival in pancreatic cancer is associated with an elevation in the quantity of CD8^+^ T lymphocytes found within the tumor tissue [35, 50, 51]. Furthermore, in prior investigations involving surgically removed samples from pancreatic cancer cases, it has been observed that the quantity of CD8^+^ T cells located in the TME exhibited a positive association with the survival rate of patients [57–60]. Early mortality related to pancreatic cancer was correlated with the percentage of CD8^+^ T cells in the peripheral region [61].

The dysfunction and exhaustion of CD8^+^ CTLs within tumors is characterized by both a decline in their ability to perform their intended functions and the presence of inhibitory receptors like PD-1, T-cell immunoglobulin and mucin domain 3 (TIM-3), and lymphocyte-activation gene 3 (LAG-3), which hinder their activity. Additionally, there are changes to their gene expression patterns. According to a model studying pancreatic cancer, the signaling of the IL-18 receptor is responsible for regulating the exhaustion of tumor-targeting CD8^+^ T lymphocytes. This occurs by activating the IL-2/STAT5/mTOR pathway [62]. Neo-adjuvant chemotherapy exhibits a reduction in the population of CD8^+^ T cells with functional exhaustion in patients affected by PDAC [63].

##### T helper (TH) cells: TH1, TH2, and TH17

*Type 1 T helper (TH1)* TH1 cells, designated as a subgroup among TH cells, emerge from the activation of naïve CD4^+^ T cells by antigen-presenting cells (APCs) under the influence of IL-12. TH1 cells strengthen the immune response of type I immune cells by promoting the activation, proliferation, and mobilization of CTLs, M1 macrophages, and NK cells. This immune reaction aids in defending the body against intracellular infections and tumor cells. These cells express the T-box transcription factor TBX21 (T-bet) and are responsible for generating anti-cancer elements such as IFN-γ, IL-2, and TNF-α [64]. Nonetheless, in the case of PDAC patients, the impact of TH1 cells remains uncertain due to the possibility that IFN-γ could induce pro-tumorigenic consequences [65]. This is because IFN-γ has the potential to elevate the expression of PD-L1 in cancer cells, thereby hindering the effectiveness of anti-tumor immunity [66]. Murine models of PDAC demonstrate that TH1 cells play a crucial role in providing defense against tumors, while in human cases, these cells are linked with extended survival [67].

Microbial dysbiosis and the disruption of epithelial barrier function are considered inducing factors in the neoplastic transformation [68, 69]. In this regard, the contribution of the microbiome to the development of pancreatic cancer and drug resistance of PDAC has been recognized [70, 71]. Bacterial ablation is associated with immunogenic reprogramming of the TME, promoting TH1 differentiation of CD4^+^ T cells [70].

*Type 2 T helper (TH2)* GATA binding protein 3 (GATA3) is responsible for defining specialized TH2 cells, known for their proficiency in combating helminths and their involvement in allergies and asthma. These differentiated cells secrete interleukin IL-4, IL-5, and IL-13. Interestingly, the differentiation of TH1 cells is hindered by TH2 cells, and vice versa. There has been an association made between the activation of DCs and the induction of TH2 responses, and it is specifically linked to the thymic stromal lymphopoietin (TSLP), which is classified as a cytokine similar to IL-7 [64]. The prevalence of GATA3^+^ TH2 cell infiltration surpasses the occurrence of T-bet^+^ TH1 cell infiltration in pancreatic cancer. The development of the disease is associated with a higher ratio of GATA3^+^/T-bet^+^ tumor-infiltrating lymphocytes (TILs) [72, 73]. IL-4 enhances the growth of pancreatic cancer cells in humans [74]. Additionally, a worse OS rate is observed in patients suffering from PDAC characterized by a higher concentration of TH2 cytokines in their bloodstream [74]. Likewise, poor survival is linked with TH2-induced inflammation in individuals suffering from pancreatic cancer [75]. However, a study reported that the inhibition of pancreas cancer growth occurs when TH2 cells enhance the anti-tumorigenic responses of macrophages and eosinophils [76].

Given the fact that ligation of Toll-like receptor 4 (TLR4) could potentially heighten inflammation in the pancreas, it can be postulated that the activation of TLR4 may play a pivotal role in the onset of pancreatic cancer. An investigation demonstrated that DCs evoke CD4^+^ TH2 cells for pancreatic antigens, thereby advancing the transition from pancreatitis to cancer. Moreover, the restraint of MyD88 is accountable for inducing these outcomes [77].

*Type 17 T helper (TH17)* The commitment to the TH17 cell lineage begins with the action of TGF-β and IL-6, and this lineage is sustained by IL-23 while being strengthened by the autocrine production of IL-21. The crucial factors RORγt and STAT3 are necessary for the development of TH17 cells and the expression of IL-17 cytokines. TH17 cells play an important role in maintaining mucosal barriers and contributing to pathogen clearance at mucosal surfaces [64]. Elevated quantities of TH17 lymphocytes have been observed in multiple types of human malignancies, such as ovarian, pancreatic, kidney, and gastric cancer [78–80]. According to several investigations, the existence of augmented levels of TH17 cells in tumor tissues or peripheral blood is linked to the progression of cancer [81, 82]. The aggressive form of the disease was found to be associated with a significant increase in the quantity of IL-17 produced by CD4^+^ TILs [83]. Conversely, alternative studies propose contrasting results and indicate that TH17 cells might possess a strong anti-tumor impact, as they are present in individuals with restricted disease or those who have survived for an extensive period of time [84, 85]. Indeed, there is an ongoing debate regarding the involvement of CD4^+^ TH17 cells in cancer [86].

IL-17A plays a significant role in PDAC by assisting in the early stages of cancer development [87, 88], controlling the characteristics of PDAC cancer stem cells (CSCs) [89], advancing tumor growth [83, 88, 90], and causing resistance to checkpoint inhibitors through the formation of NETs [91]. Additionally, recent studies have revealed that IL-17A affects the transcriptome of cancer-associated fibroblasts (CAFs) [92]. Prominently, the induction of CAFs that are inflammatory is promoted by T cells that produce IL-17A, thus contributing to the progression of PDAC [93]. The promotion of tumorigenesis is facilitated by the upregulation of B7-H4 through IL-17/IL-17 receptor signaling in the pancreatic epithelium [94]. These findings accentuate the role of TH17 cells in favor of pancreatic cancer progression.

Contrary to the aforementioned findings, there exist findings demonstrating that TH17 cells act against tumor cells. Enhancing survival in a murine model of pancreatic cancer is observed through the promotion of TH17 cell development within the TME [95]. All in all, the role of TH17 and IL-17A in pancreatic cancer is not yet fully understood, with evidence suggesting both pro-tumorigenic and anti-tumorigenic effects. Further research is needed to elucidate the mechanisms through which IL-17A influences pancreatic cancer progression and to determine the potential therapeutic implications of targeting IL-17A in this disease.

##### Regulatory T cells (Tregs)

Tregs express CD4, CD25, and a chief transcription factor, called forkhead box P3 (FOXP3). The prevention of autoimmune disorders, the limitation of chronic inflammatory diseases, and the maintenance of peripheral tolerance all hinge upon Tregs. Furthermore, Tregs play a crucial role in the tumor environment, influencing cancer progression and immune responses [96]. Tregs can exert their suppressive effects through various mechanisms, whether by direct contact or independently. These mechanisms include: The production of suppressive cytokines such as TGF-β, IL-10, and IL-35. The engagement of inhibitory immune checkpoints and enzymes, such as cytotoxic T-lymphocyte-associated protein 4 (CTLA-4), PD-1, LAG-3, TIM-3, T cell immunoreceptor with Ig and ITIM domains (TIGIT), CD39, CD73, and IDO. The induction of direct cytotoxicity through the release of perforin/granzyme. The disruption of T effector cell activity through metabolic alterations, specifically IL-2 consumption. The initiation of a tolerogenic environment by inducing tolerogenic DCs, which then facilitates T cell exhaustion [97–99].

In the peripheral blood and TME, individuals suffering from pancreatic cancer exhibit an increased frequency of Tregs [100, 101]. Tregs play a part in controlling the immune response as PDAC advances from a premalignant state to a cancerous stage. The presence of elevated Tregs is linked to a more unfavorable prognosis in PDAC [102]. Tregs possess the ability to restrict the proliferation and immunogenicity of DCs in pancreatic cancer. Additionally, the stimulation of anti-tumor immunity in pancreatic cancer is achieved by diminishing Tregs in a manner that relies on CD8^+^ -activated T-cells [103]. Contrariwise, the depletion of Tregs shapes the TME, leading to an acceleration of pancreatic carcinogenesis [104]. There is an expansion of pro-inflammatory and immunosuppressive Tregs which simultaneously express RORγt and FOXP3 [105]. The underlying rationale for this dual functionality can be elucidated as follows: the presence of plasticity within the pancreatic cancer microenvironment enables the Tregs to exhibit the characteristic phenotype of TH17 cells.

#### Role of NK cells in TME

NK cells, which are a distinct type of immune cell found in the innate immune system, are believed to play a role in monitoring and controlling tumor growth and tumor immunosurveillance [106, 107]. Both preclinical and clinical studies have demonstrated a link between decreased NK cell activity and an increased susceptibility to cancer as well as a higher chance of cancer spread and metastasis [108–110]. Researchers have identified several mediators, including indoleamine 2, 3-dioxygenase (IDO), matrix metalloproteinases (MMPs), TGF-β, and IL-10, that contribute to immune suppression in pancreatic cancer, impeding the ability of NK cells to recognize and eliminate tumor cells [28].

The survival of individuals with PDAC was found to be positively correlated with the relative frequency of NK cells in their blood. However, PDAC-associated NK cells demonstrated lower cytotoxicity compared to those of healthy participants [111]. Patients with PDAC were observed to have diminished expression of NKG2D, NKp46, and NKp30 on their peripheral NK cells, which was connected to the patient’s stage and histological grade [112]. Furthermore, the decreased expression of CD96 and CD226 (key regulators of NK cell function) on NK cells was linked to the development of cancer in PDAC patients [113]. Additionally, the evasion of NK cells in human pancreatic cancer is associated with the expression of Igγ-1 chain C region (IGHG1). Mechanistically, the presence of IGHG1 suppressed the cytotoxic activity of NK cells by inhibiting antibody-dependent cellular cytotoxicity (ADCC) [114]. Moreover, impaired localization resulting from the absence of CXCR2 and impaired tumor cytotoxicity contributed to NK cell immune evasion in patients with pancreatic cancer [115]. The function of NK cells is inhibited in the microenvironment of human PDAC by activated pancreatic stellate cells [116]. In pancreatic cancer, the orchestration of anti-tumor immune responses through CXCL8 (IL-8) by radiotherapy is reliant on NK cells. In xenografted mice, the use of high-dose radiotherapy in conjunction with adoptive NK cell transfer resulted in enhanced tumor control compared to using either treatment alone, indicating that combining NK cells with radiotherapy is a logical approach for cancer therapy [117]. Inhibiting the protein growth arrest specific 6 (Gas6), which is generated by tumor-associated macrophages (TAMs) and CAFs within the TME of PDAC, reverses the process of epithelial-mesenchymal transition (EMT) and enhances the activation of NK cells [118].

#### Role of DCs in TME

DCs, which are crucial for effective anti-tumor T cell responses, are scarce in the pancreatic tumor environment and are usually found at the tumor edges [119]. An increased presence of type-1 conventional DCs (cDC1s) within the entire tumor area and the tumor stroma was notably linked to improved disease-free survival (DFS). Furthermore, a rise in the number of cDC2s infiltrating the tumor’s epithelial layer was associated with enhanced DFS and OS [120]. Furthermore, patients with pancreatic cancer have been shown to have lower levels of DCs in their blood [121]. Interestingly, higher levels of circulating DCs are linked to better survival rates in these patients [121, 122]. Additionally, the surgical removal of the pancreatic tumor has been found to enhance the function of blood DCs, suggesting that the tumor itself may influence immune function [123, 124].

Cytokines originating from tumors, including TGF-β, IL-10, and IL-6, have been identified as factors that inhibit the survival and growth of DCs [125]. MDSCs generate nitric oxide (NO) and obstruct the activation of DCs [126]. In pancreatic tumors, T-cell dysfunction is common, and improving DC-mediated T-cell activation could be key for treatment. Dysfunction of cDC1s in PDACs leads to unresponsiveness to checkpoint immunotherapy. A study of 106 samples from PDAC patients showed decreased levels of circulating cDC2s, which was linked to poor prognosis. Elevated levels of IL-6 in PDAC patients were found to negatively impact DC numbers and differentiation. This suggests that inflammatory cytokines suppress DCs, impairing antitumor immunity [127].

DCs control T cells via cross-priming (cross-presentation). It is an open question in PDAC whether boosting the cross-priming capacity of DCs can enhance the T cells’ anti-tumor activity and remodel the TME. In the process of cross-priming, foreign antigens are absorbed by APCs, processed, and then displayed on MHC-I. This sequence of events ultimately triggers the activation of CD8^+^ T-cell responses [128]. Research has shown that the cross-priming of cDC1 is not only necessary for starting CD8^+^ T-cell responses as tumors progress, but it also has a pivotal role in the reactivation of tumor-specific CD8^+^ T cells through immunotherapy, leading to tumor shrinkage [129]. However, during the development of pancreatic cancer, the maturation of cDC1 is increasingly and universally hindered [130], impairing cross-presentation machinery. As a first proof of concept, a study tested whether cross-presentation by DCs could activate pancreatic tumor-specific CD8^+^ T cells in vaccinated pancreatic cancer patients. The process of in vivo cross-priming leads to the activation of mesothelin (MSLN)-specific CD8^+^ T cells in patients who received a vaccine for allogeneic pancreatic tumors. Also, the vaccine recruits DCs that cross-prime and generate MSLN-specific CD8^+^ T cells, which are capable of destroying tumor cells expressing MSLN [131]. All in all, the immunosuppressive pancreatic TME leads to the disruption of the cross-priming ability of DCs. Thus, finding solutions to reinvigorate the DCs to cross-prime tumor antigens paves the way for developing novel therapies that boost the anti-tumor immune response mediated by CD8^+^ T cells.

#### Role of macrophages in TME

Monocytes in circulation are drawn towards the TME and transform into macrophages, called TAMs, when exposed to cytokines, chemokines, and various stimuli, including high levels of concentration of hypoxia and lactic acid [132–134]. Several studies revealed that the CCL2/CCR2 and CXCL17/CXCR8 axes are involved in recruiting monocytes into the site of inflammation and tumor [135, 136]. TAMs display diverse polarization states called functional states. A wide range of TAM subpopulations has been discovered and is continuously growing. They are commonly classified as “M1” and “M2” macrophages. M1 macrophages, as typically described, generate pro-inflammatory cytokines with mainly anti-neoplastic impacts, whereas M2 macrophages produce anti-inflammatory signals that potentially accelerate tumor development [137–140]. The presence of tissue-resident macrophages in PDAC is a result of their origin from embryonic hematopoiesis, and these macrophages play a crucial role in advancing the progression of tumors [141].

A range of scientific investigations on various tumor types, including pancreatic cancer, have demonstrated a contrary association between the invasion of TAMs and the prognosis of patients [133, 142–144]. Multiple research groups have confirmed that TAMs are responsible for fostering immunosuppression, angiogenesis, and the growth of tumors in mouse models of PDAC. Their mechanism involves the release of growth factors like vascular endothelial growth factor (VEGF), cytokines, and proteases [145–149]. Within the PDAC microenvironment, the presence of granulocyte–macrophage colony-stimulating factor (GM-CSF) and lactate plays a crucial function in the polarization of TAMs, which are molecules discharged from cancer cells in a manner reliant on a mutant KRAS. A study has shown that TAM gene expression and metabolism are adversely affected by GM-CSF, disrupting their regulation through PI3K-AKT pathway signaling [150]. Collagen turnover in pancreatic cancer causes metabolic reprogramming of TAMs, leading to the promotion of fibrosis and extracellular matrix (ECM) remodeling [151].

The effectiveness of treatment in PDAC can be significantly reduced by TAMs. TAMs impact the function of cytidine deaminase, which is a critical enzyme in the metabolism of gemcitabine. This, in turn, leads to resistance to gemcitabine-based treatments in animal models of PDAC [152]. In mice models of PDAC, the suppression of C–C chemokine receptor type 2 (CCR2) promotes T-cell infiltration, enhances the efficacy of radiotherapy and chemotherapy, and diminishes metastasis by preventing the migration of monocytes to the TME [153–155]. Also, the combination of CCR2 and CXCR2 inhibitors can interrupt the accumulation of CCR2^+^ TAMs and CXCR2^+^ tumor-associated neutrophils (TANs) in the TME and enhance the effectiveness of chemotherapy in treating PDAC [147]. Moreover, the expression of CXCR2 is also reported on TAMs [156, 157]. For example, in Pten-null prostate tumors, CXCR2^+^ TAMs are abundant. Activating CXCR2 shifts these macrophages to an anti-inflammatory state, but blocking CXCR2 with a selective antagonist reprograms them to a pro-inflammatory state [156]. Also, in pancreatic cancer mouse models, CXCR2^+^CD68^+^ macrophages (M2 phenotype) are recruited to the TME by tumor-derived CXCL8, where they contribute to local immunosuppression, thereby reducing the effectiveness of PD-1 blockade therapy [157]. Thus, blocking the CXCR2 pathway offers a therapeutic option for enhancing cancer immunotherapy in PDAC. In a study, the tumor burden, M2 macrophage polarization, and migration are reduced, and the response to immunotherapy with anti-PD-1 is enhanced by ladarixin, a CXCR1/2 dual-inhibitor [158]. In pancreatic cancer models, the reprogramming of TAMs through colony-stimulating factor 1 (CSF1)/colony-stimulating factor 1 receptor (CSF-1R) blockade enhances the response to T-cell checkpoint immunotherapy [159].

#### Role of myeloid-derived suppressor cells (MDSCs) in TME

MDSCs, a diverse group of immature myeloid cells, are commonly categorized into two types: monocytic (M-MDSC) and granulocytic (polymorphonuclear [PMN]-MDSC). M-MDSCs closely resemble monocytes in terms of their phenotype and physical characteristics, while PMN-MDSCs are equivalent to neutrophils. MDSCs play a paramount role in cancer progression by promoting immunosuppression, shaping the TME, and facilitating the formation of pre-metastatic niches. Within the microenvironment of human tumors, MDSCs are abundant, and typically, PMN-MDSCs make up more than 80% of all MDSCs associated with tumors [160, 161]. Furthermore, in the circulation of the portal vein, the survival and immunoresistance of PDAC circulating tumor cells are supported by influencing the differentiation of MDSCs [162].

The levels of MDSCs in human PDAC are associated with the stage of cancer [143, 163, 164]. GM-CSF, produced by tumor cells at the early stages of cancer, plays a crucial role in the recruitment and differentiation of MDSCs, as confirmed by studies on genetically modified mice [165, 166]. CD73 causes the acceleration of pancreatic cancer pathogenesis by inducing T cell suppression through GM-CSF/MDSC [167]. Additionally, the receptor for advanced glycation end products (RAGE) facilitates the accumulation of MDSCs and promotes pancreatic carcinogenesis [168]. High expression levels of Yes-associated protein (YAP) or MDSC-associated genes indicate poor survival in PDAC patients. YAP expression levels are significantly correlated with a gene signature associated with MDSCs in primary human PDAC [169]. Following the mutation of KRAS, the transcription regulator YAP, as a downstream molecule of the oncogenic KRAS, plays a crucial role in the neoplastic development leading to PDAC [170]. The interaction between YAP/TAZ (downstream effectors of the Hippo pathway) and TEAD proteins facilitates the cancer-promoting functions of YAP. Thus, small-molecule inhibitors like GNE-7883 and IAG933, which block the interactions between YAP/TAZ and TEAD, can disrupt oncogenic YAP/TAZ signaling in RAS-altered tumors like PDAC [171, 172]. Within the PDAC microenvironment, CD200, a regulator of myeloid cell function, is upregulated. Moreover, MDSCs from PDAC patients show increased expression of the CD200 receptor. CD200 expression may regulate the development of MDSCs in the microenvironment of PDAC [173].

MDSCs control the inhibition of tumor activity in CD4^+^ and CD8^+^ T lymphocytes. T-cell activation is repressed by PD-L1, which is upregulated by MDSCs through the PD-L1/PD-1 interaction [174]. Furthermore, in an interleukin-10 (IL-10)-dependent manner, MDSCs can limit T-cell activity by promoting the growth of immune-suppressive regulatory T cells (Tregs) through the release of TGF-β and interferon-gamma (IFN-γ) [175, 176]. MDSCs play a significant role in both primary and acquired resistance to cancer immunotherapy [177]. In PDAC, reducing MDSCs enhances the accumulation of stimulated CD8^+^ T lymphocytes within the tumor, leading to cell death in tumor epithelial cells and remodeling of the tumor stroma [178]. Strategic MDSC targeting has been observed to effectively revitalize cytotoxic anti-tumor responses in PDAC cases. This mechanism induces the repolarization of TAMs and instigates the activation of the inflammasome machinery, thereby leading to the production of IL-18. The subsequent upregulation of IL-18 notably amplifies the functional capabilities of T-cells and NK cells within the TME [179]. In conclusion, targeting MDSCs presents a promising approach to the treatment of PDAC, and it has shown positive effects in revitalizing cytotoxic anti-tumor responses and enhancing the functional capabilities of T cells and NK cells. Therefore, further research into MDSC targeting could potentially lead to more effective therapeutic strategies for PDAC.

#### Role of neutrophils in TME

Neutrophils act as the first line of protection in the body against infection and respond to a broad range of pro-inflammatory signals and alarmins, such as cancer cells. These cells possess adaptability or plasticity, allowing them to adjust their actions when faced with different inflammatory triggers [180]. Because of the inflammatory state of the TME in PDAC, tumor cells secrete pro-inflammatory substances like tumor necrosis factor-alpha (TNF-α) and IL-12, causing the recruitment of neutrophils to the location of the tumor [181]. Factors secreted by tumor cells can attract neutrophils. Neutrophils can be drawn in by IL-1, CD200, CXCR2 ligands (like CXCL1 [in human and mouse], CXCL2 [in human and mouse], CXCL5 [in human], and CXCL8 [in human]) [182], GM-CSF (in human), granulocyte colony-stimulating factor (G-CSF; in human and mouse), and various other substances. These factors are released by tumor cells to attract neutrophils [182–184]. There exists a notable correlation between shortened survival and worse prognosis in patients with PDAC and increased quantities of neutrophils infiltrating the TME [60, 185].

The roles of neutrophils in the TME vary depending on their polarization states, either promoting or suppressing cancer growth. TME attracts TANs through the action of cytokines and chemokines. TANs can be categorized based on their activation and cytokine profile, which determines their impact on the growth of tumor cells. N1 TANs exhibit a beneficial effect on tumor suppression either through direct cytotoxicity or indirect means. N2 TANs, on the other hand, promote immunosuppression, tumor expansion, angiogenesis, and metastasis by causing DNA instability and releasing cytokines and chemokines [186]. Recently, a new type of TANs called T3 neutrophils has been discovered. These T3 neutrophils stimulate angiogenesis, thus improving the ability of pancreatic tumors to survive in low-oxygen and nutrient-deficient environments [187]. Identifying the plasticity of N1/N2 neutrophils has been deemed a critical prognostic marker, potentially demonstrating TME and immune evasion in PDAC patients [188]. Neutrophils with anti-tumor properties can directly eliminate tumor cells through the production of reactive oxygen and nitrogen species. Additionally, they have the ability to activate T cells and attract pro-inflammatory M1 macrophages. Conversely, neutrophils that aid tumor development secrete MMP-9, facilitating the growth of new blood vessels and the dissemination of tumor cells. These neutrophils can also hinder the function of NK cells while recruiting anti-inflammatory M2 macrophages and Tregs. Further, suppressor neutrophils, referred to as PMN-MDSCs, as well as other pro-tumoral neutrophils, impede the activity of CD8^+^ T cells [24, 180]. The growth of pancreatic cancer is reduced and the effectiveness of ICB treatment with anti-PD-1 is enhanced through the inhibition of TANs by lorlatinib [189]. The metastasis of pancreatic cancer is facilitated by neutrophils that infiltrate as a result of chemotherapy. This is achieved through the activation of the Gas6/AXL signaling pathway [184].

Neutrophils differentiate themselves from other immune cells by producing neutrophil extracellular traps (NETs), consisting of DNA fibers and proteolytic enzymes released to counteract infections [190]. Nevertheless, recent studies have suggested that NETs might contribute to cancer metastasis. By examining a PDAC mouse model, researchers investigated the effects of DNase I, a NET inhibitor, and observed a reduction in liver metastasis [191]. In the PDAC milieu, neutrophil recruitment and NETosis are triggered by IL-17 [91]. The activation of the IL-1β/epidermal growth factor receptor (EGFR)/extracellular-signal-regulated kinase (ERK) pathway is prompted by NETs, resulting in the promotion of migration, invasion, and EMT of pancreatic cancer cells [192].

#### Role of B lymphocytes in TME

A study found that a high density of B cells within tertiary lymphoid tissues of human PDAC is associated with longer survival rates, germinal center immune signature, and CD8^+^ TILs infiltration [193]. In the TME of PDAC, the predominant B cells are plasma cells and memory B cells, which exhibit high levels of CD27 expression. However, numerous studies have discovered that the upregulation of CXCL13, triggered by IL-1β and type I interferons (IFNs-I), leads to an increased influx of regulatory B cells (Bregs) that perform immunosuppressive activities [194–196]. Bregs can activate STAT3 signaling within themselves and CD8^+^ T cells via IL-35. This activation leads to two distinct effects: firstly, the transcriptional regulator BCL-6 experiences an increase in naive B cells, which interferes with the transformation of B cells into plasma cells; secondly, the operational capacity of CTLs is suppressed [197, 198]. Recent research discovered that the resistance to the stimulator of interferon genes (STING) agonists in PDAC is attributed to the induction of IL-35^+^ B cell proliferation. The systemic application of anti-IL-35 and STING agonist (cyclic guanosine monophosphate-adenosine monophosphate [cGAMP]) can work together to suppress the amplification of Bregs and boost the effectiveness of NK cells [199]. A clinical trial showed that ibrutinib (a Bruton tyrosine kinase inhibitor) plus nab-paclitaxel/gemcitabine did not improve OS or progression-free survival (PFS) for patients with PDAC [200].

### The role of non-immune cells

Within the microenvironment of pancreatic tumors, there exists a variety of non-immune cells. This section delves into a discussion about the most significant among them.

#### Pancreatic cancer stem cells (PCSCs)

PCSCs are a subset of cancer cells that exhibit stem cell-like characteristics, including the ability to self-renew and initiate tumorigenesis. They are believed to contribute to the initiation, metastasis, and recurrence of PDAC, and are also responsible for resistance to chemotherapy and radiation. PCSCs express several markers, including CD133, CD24, CD44, microtubule-associated doublecortin-like kinase 1 (DCLK1), CXCR4, epithelial-specific antigen (ESA), OCT4, nestin, and ABCB1 [201, 202]. In PDAC, stem cells display unusual activation of multiple signaling pathways that are generally active in embryonic growth. This irregular signaling via mechanisms such as Hedgehog, Wnt, Notch, JAK-STAT, Nodal/Activin, and Hippo enables PCSCs to preserve their self-renewal ability, develop resistance to chemotherapy and radiation, enhance their capacity to induce tumors, and spread to other parts of the body [202]. A specific subpopulation of CSCs, identified by CD133 and CXCR4 markers, is crucial for tumor metastasis in human pancreatic cancer. Depleting this subpopulation can significantly reduce metastasis. Modulating the CXCL12/CXCR4 axis could be a potential strategy to inhibit CSC metastasis [203]. The E2F1/4-pRb/RBL2 axis, which undergoes deregulation following a KRAS mutation, is instrumental in maintaining equilibrium among signaling pathways controlling stem cell-like characteristics of CSCs. This axis governs the production of Wnt ligands, thereby managing the self-renewal, resistance to chemotherapy, and invasive nature of PCSCs, along with the proliferation of fibroblasts [204]. This axis might be a therapeutic target for eradicating PCSCs.

#### Mesenchymal stem/stromal cells (MSCs)

MSCs are a heterogeneous group of progenitor cells that transform into tumor-associated mesenchymal stem cells (TA-MSCs) within TME, influencing tumor growth, metastasis, angiogenesis, and treatment responses through the secretion of various factors, and their immunosuppressive properties could be targeted to enhance anti-tumor immunity [205]. First of all, TA-MSCs can release CCL2, CCl7, and CCL12 to recruit monocytes, macrophages, MDSCs, and neutrophils [206]. They also produce CXCL9 [207], CXCL10 [207], CXCL11 [207], inducible nitric oxide synthase (iNOS) [207], and IDO [208], resulting in the inhibition of effector T cells. Mechanistically, TA-MSCs produce large amounts of pro-metastatic and pro-tumor factors such as neuregulin-1 [209], VEGF [210], bone morphogenetic proteins [211], TGF-β [212], CCL5 [213], CXCL10 [214], CXCL12 [215], CD81-positive exosomes [216], and MMPs [217]. Also, they can adjust tumor cell’s response to chemotherapy by generating factors like polyunsaturated fatty acids [218], PDGF [219], hepatocyte growth factor [220], NO [221], and exosomes carrying these factors and microRNAs [222, 223]. In patients with pancreatic cancer, the presence of MSCs in the peripheral blood is notable as they are thought to migrate to the tumor mass [224]. Evidence suggests that a significant portion of CAFs may originate from MSCs, which can differentiate and express CAF markers, such as vimentin and FAP when exposed to conditioned media from various human cancer cell cultures like pancreatic cancer [225]. In a pancreatic cancer tumor model, VEGF is secreted by bone marrow mesenchymal stem cells (BM-MSCs) that are co-injected with tumor cells, which aids in the promotion of tumor angiogenesis [210]. TA-MSCs can produce NO, which induces resistance to etoposide in pancreatic tumor cells and forms a positive feedback loop with IL-1β, contributing to chemotherapy resistance [221].

#### Cancer-associated fibroblasts (CAFs)

CAFs are a hodgepodge and heterogeneous group of stromal cells that produce ECM proteins. These cells, typically spindle-shaped, express activated fibroblast markers like fibroblast activation protein (FAP) and α-smooth muscle actin. They are associated with various tumor-promoting activities, including tumorigenesis, angiogenesis, immunosuppression, and metastasis [226, 227]. CAFs in PDAC can originate from diverse cells like adipocytes, pericytes, bone marrow-derived macrophages, endothelial/epithelial cells, mesothelial cells, MSCs, resident tissue fibroblasts, and pancreatic stellate cells (PSCs) [228]. In PDAC stroma, CAFs interact with cancer cells through both direct cell-to-cell and paracrine mechanisms. CAFs are heterogeneous and include three subtypes: myofibroblastic, inflammatory, and antigen-presenting. Myofibroblastic CAFs are induced by cancer cells through TGF-β, and they create a mechanical barrier that can both promote and inhibit tumor growth. Inflammatory CAFs, located away from the tumor cells, are reprogrammed by IL-1 to generate cytokines and chemokines (like IL-6), which further stimulate cancer growth. Lastly, antigen-presenting CAFs express MHC class II molecules and modulate the immune cells in the stroma. These diverse interactions contribute to the complex dynamics of the PDAC stroma [12]. In the pancreatic environment, CAFs play a significant role in creating an immune-suppressive milieu by releasing substances like prostaglandin E2 (PGE2), IL-1, IL-6, CXCL2, CXCL12, and CXCL8 [35, 229–231]. Not only do these fibroblasts attract and control immune-suppressing cells, but they also hinder the anti-cancer activities of CD8^+^ T cells by increasing the expression of inhibitory immune checkpoints [230]. Recently, a study identified three distinct metastasis-associated fibroblasts (MAFs) populations, with the generation of pro-metastatic myofibroblastic-MAFs (myMAFs) being critically dependent on macrophages. These myMAFs are induced through a STAT3-dependent mechanism and in turn promote an immunosuppressive macrophage phenotype, inhibiting cytotoxic T-cell functions. Blocking STAT3 pharmacologically or depleting it in myMAFs restores an anti-tumor immune response and reduces metastasis, providing potential targets to inhibit PDAC liver metastasis [232].

#### Pancreatic stellate cells (PSCs)

Approximately 7% of pancreatic cells are made up of PSCs, which are located in both the exocrine and endocrine regions of the pancreatic tissue. The interaction between PSCs and pancreatic cancer cells promotes tumor progression. Mechanistically, PSCs release several growth factors/mediators (such as insulin-like growth factor 1 [IGF-1], basal fibroblast growth factor [bFGF], platelet-derived growth factor [PDGF], stromal cell-derived factor 1 [SDF-1], and ECM proteins) and MMPs, which provoke the proliferation, migration, and invasion of pancreatic tumor cells. In response, pancreatic cancer cells produce TGF-β1, PDGF, and VEGF, which in turn stimulate PSCs to increase the migration and proliferation of CAFs and the production of ECM [233, 234]. Indeed, a key characteristic of PDAC is a desmoplastic reaction, seen in both primary and metastatic tumors. This reaction is caused by the activation of PSCs, by cancer cells, leading to fibrosis around the tumor [235, 236]. This fibrosis (also known as desmoplasia) forms a mechanical barrier around the tumor cells, hindering proper vascularization, limiting the effectiveness of chemotherapy, and resulting in poor immune cell infiltration [237]. PSCs serve as a significant source of MMP-2 and they hasten the advancement of the tumor in a murine xenograft model [238]. Also, TGF-β1 secreted by PSCs promotes stemness and tumourigenicity in pancreatic cancer cells through L1CAM downregulation [239]. Overall, PSCs are linked to ECM production and remodeling, intra-tumoral hypoxia, resistance/barrier to chemotherapy, proliferation, invasion, migration, reduced apoptosis, angiogenesis, immune suppression, and pain factors [234].

#### Endothelial cells

PDAC often has abnormal blood and lymphatic vessels, leading to a hostile microenvironment characterized by high acidity, hypoxia, aberrant metabolism, and immune evasion. In response, tumors stimulate angiogenesis, promoting tumor growth and metastasis [46, 240]. Studies reveal that high expression of the endothelial cell marker CD31 and genes involved in vascular stability correlate with better prognosis and improved survival in PDAC [241, 242]. This suggests that a subset of patients with highly vascular PDAC may benefit from antiangiogenic therapies [242].

Inadequate vasculature in tumors restricts nutrient, oxygen, and leukocyte delivery, leading to hypoxia in PDAC. Hypoxia-inducible factor 1α (HIF-1α) is stabilized in poorly vascularized PDAC tumors [243], activating genes crucial for metabolism, angiogenesis, cell survival, and inflammation [244]. Elevated HIF-1α levels are linked to poor prognosis in many cancers [244]. However, in PDAC, HIF-1α deletion accelerates tumor growth, facilitated by infiltrating B cells, demonstrating PDAC’s resilience and complex redundancies that support disease progression [245].

Lymphatics, in addition to blood vessels, play a crucial role in the progression of PDAC. They serve as a major pathway for leukocytes to transport tumor antigens to lymph nodes and for cancer cells to spread, often resulting in worse survival outcomes [46, 246, 247]. Chemokines play a role in lymphangiogenesis and cell migration, with lymphatic endothelial cells secreting CCL21 to attract DCs and tumor cells expressing CCR7 potentially using this mechanism for dissemination [248]. Likewise, CXCL12 produced in lymph nodes may attract cancer cells or leukocytes expressing CXCR4 [249].

## Immunotherapeutic approaches in pancreatic cancer treatment

**Fig. 3:**
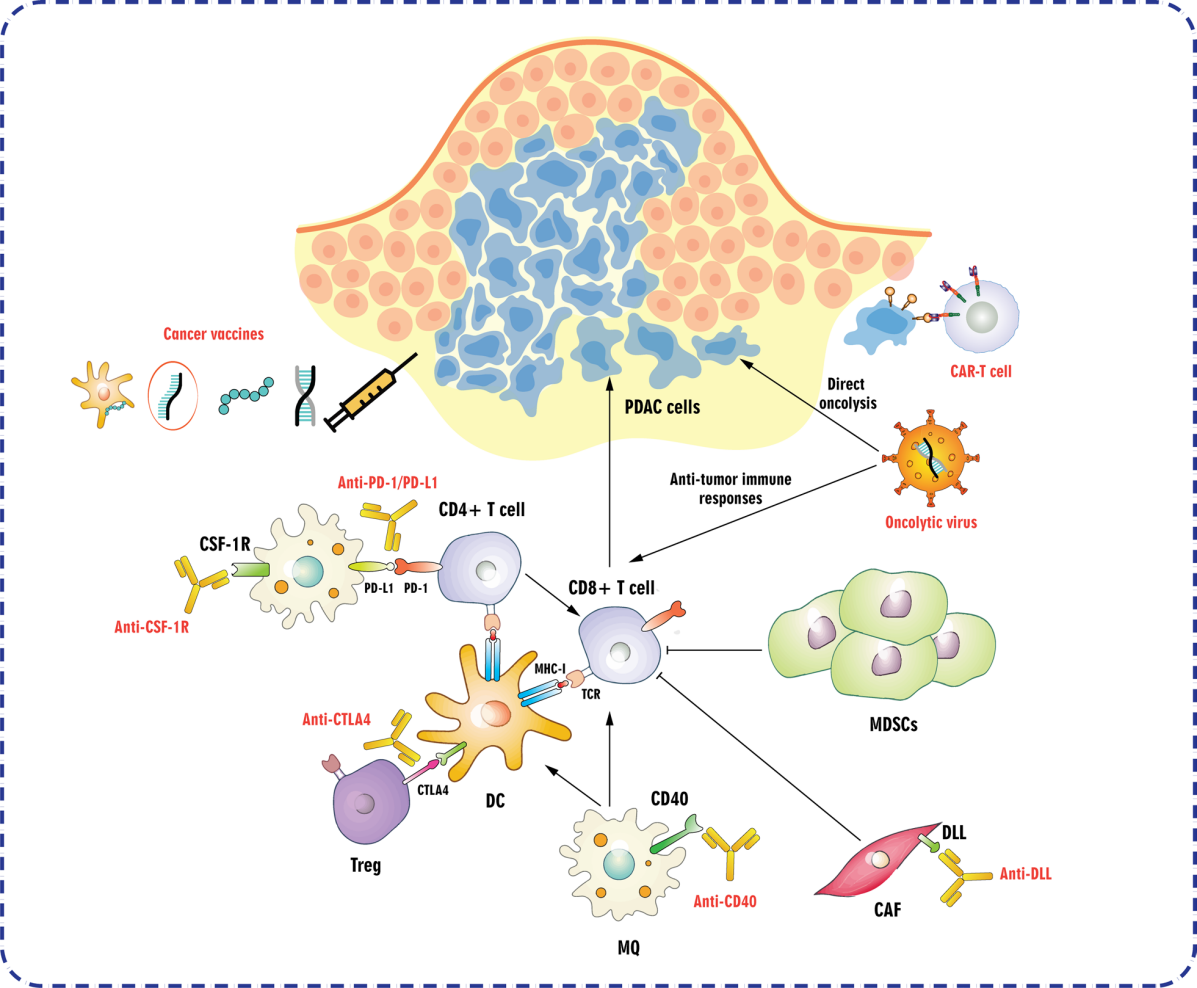
Immunotherapeutic strategies in pancreatic cancer treatment. The immune response to pancreatic ductal adenocarcinoma (PDAC) is guided by antigen-presenting machinery involving dendritic cells (DCs), inflammatory macrophages, and CD4^+^ helper T cells, leading to the activation of CD8^+^ cytotoxic T cells to eliminate the cancer. However, regulatory T cells (Tregs) and suppressor cells can inhibit this response, creating an immunosuppressive tumor microenvironment. Various strategies have been suggested to counteract these inhibitory pathways. CAF: Cancer-associated fibroblast; CAR: Chimeric antigen receptor; CSF-1R: Colony-stimulating factor 1 receptor; CTLA4: Cytotoxic T-lymphocyte associated protein 4; DLL: Delta-like ligand; MDSC: Myeloid-derived suppressor cell; MHC: Major histocompatibility complex; MQ: Macrophage; PD-1: Programmed cell death protein 1; PD-L1: Programmed death-ligand 1; TCR: T cell receptor

Pancreatic cancer is classified as non-immunogenic and immunologically cold since it does not effectively react to commonly employed ICIs such as anti-PD-1 and anti-CTLA-4. This resistance is partly caused by the immunosuppressive circumstances within the TME. In other words, although ICB has achieved explosive success, PDAC has shown limited response to ICB treatment alone. Research on using ICB alone or in combination with anti-PD-1 and anti-CTLA-4 antibodies has yielded overall response rates (ORRs) of 0% and 3%, respectively [250]. In this part, we will delineate immunotherapeutic strategies such as OVT, adoptive cell transfer therapy, ICB, cancer vaccine, and immunotherapies targeting myeloid cells (Fig. 3).

### Oncolytic virus therapy (OVT)

OVT represents an innovative form of immunotherapy where an oncolytic virus, upon infiltrating and lysing a cancerous cell, initiates an immune reaction within the patient by discharging tumor antigens into the circulatory system [251]. Oncolytic viruses possess desirable qualities and specificity that make them an attractive strategy for treatment. Research is currently underway, exploring and utilizing diverse oncolytic DNA and RNA viruses for the treatment of different cancer forms. Their ability to invade cancer cells is made possible by the genetic composition of these viruses [252].

Talimogene laherparepvec (T-VEC or OncoVEXGM-CSF), a Herpes simplex virus (HSV), has become the inaugural oncolytic virus approved by the US Food and Drug Administration (FDA) for the treatment of melanoma. The T-VEC virus harbors the genetic integration of the GM-CSF gene. T-VEC exhibited remarkable lytic properties when tested against various tumor cell lines, encompassing pancreatic cancer cells [253, 254]. Furthermore, both NV1020 (r7020) and G207, two distinct herpes simplex oncolytic viruses, effectively invade and annihilate human pancreatic cancer cells in vitro and in vivo [255]. HF10 is a virus that has originated from HSV-1 and has experienced an unexpected mutation. This particular virus has the ability to substantially combat tumors without causing any damage to healthy tissue. The treatment of locally advanced pancreatic cancer involves the secure administration of HF10 through direct injection, alongside erlotinib and gemcitabine [256]. The anti-tumor response and apoptosis are enhanced in pancreatic cancer when an H-1 oncolytic parvovirus is combined with a hypoxia-inducible factor (HIF)-1α inhibitor, resulting in increased effectiveness [257].

VCN-01, a type of oncolytic adenovirus, has been specifically designed to reproduce within cancer cells that possess a faulty RB1 pathway. Moreover, it has the ability to generate hyaluronidase, which serves to expedite the spread of the virus within the tumor. Additionally, it facilitates the migration of both chemotherapy medications and immune cells into the tumor. VCN-01 exhibited augmented anti-cancer properties when administered in conjunction with chemotherapy to animals with PDAC. Remarkably, the hyaluronidase produced by VCN-01 effectively obliterated the tumor stroma, thereby bolstering the transport of various therapeutic drugs such as chemotherapy and therapeutic antibodies [258]. A clinical experiment exhibited that it is feasible to administer VCN-01 through an intravenous route for the treatment of patients suffering from PDAC and this administration method is associated with adverse events (AEs) that can be predicted and controlled. Intravenous VCN-01 has exhibited a positive tolerability profile [259]. These results establish a helpful bedrock for the future use of OVT in pancreatic cancer immunotherapy. Furthermore, several clinical trials are underway to evaluate the efficacy of various oncolytic virus-oriented therapies in pancreatic cancer. A phase I/II trial demonstrated that the combination of intratumoral injections of LOAd703, an oncolytic adenovirus with transgenes encoding trimerized, membrane-bound (TMZ)-CD40L and 4-1BB ligand, with standard nab-paclitaxel/gemcitabine chemotherapy was both safe and feasible for patients with unresectable or metastatic PDAC. The treatment met the target response rate at the highest dose level, with an ORR of 44% and a disease control rate of 94% (NCT02705196) [260]. Moreover, a study found that the combination of pelareorep and pembrolizumab showed modest efficacy in unselected patients, with a clinical benefit rate of 42% among the 12 patients. Notably, the treatment led to significant immunological changes, including a decrease in VDAC1 expression in peripheral CD8^+^ T cells and on-treatment peripheral CD4^+^ Treg levels in patients who responded to the treatment (NCT03723915) [261]. The efficacy of talimogene laherparepvec (T-VEC), administered endoscopically, will be assessed in a clinical trial for the treatment of locally advanced or metastatic pancreatic cancer that is refractory to at least one chemotherapy regimen (NCT03086642).

A study demonstrates promising findings for a new technology called ONCOTECH, which combines oncolytic adenoviruses (OAs) with T cells to enhance the delivery of viruses to tumors. The engineered OAs target the immune checkpoint protein PD-L1. In mouse models of PDAC, ONCOTECH displayed a notable increase in OAs within tumor cells, resulting in a significant decrease in PD-L1 expression and better survival rates. In summary, ONCOTECH has the potential to be a successful approach in combining virotherapy and cell therapy for cancer treatment [262].

### Adoptive cell transfer therapy

Adoptive cellular therapy, which is a type of immunotherapy, holds promise for cancer patients. By utilizing the patient's immune cells, such as T cells, this technique endeavors to combat the disease. These immune cells are frequently obtained, replicated, and altered to augment their efficiency in directing their focus on cancer. The progress made by the FDA in granting approval to CAR T-cell therapy for certain blood cancers has greatly propelled this area of medical research. Modified T cells possess the ability to discern tumor cells through their unique molecular features [263]. In the subsequent discussion, we shall elucidate and analyze these various immunotherapeutic approaches.

#### Tumor-infiltrating lymphocyte (TIL) therapy

TILs, which are mononuclear cells naturally infiltrating the TME, can also be known as immune cells present at the tumor site. TIL therapy remains a hopeful treatment approach whereby the patient's TILs are utilized following the surgical extraction of the cancerous growth, followed by the cultivation of these cells outside the body and subsequent reinfusion back into the patient [264–266]. Successful techniques for increasing the production and reactivity of TILs encompass inhibiting the PD-1 receptor, stimulating the CD137 receptor (4-1BB), and augmenting CD8^+^ T cell levels [267]. According to a study, it was found that functional expanded TILs from tumors in the pancreas possess the capability to identify antigens associated with pancreatic cancer [267]. Based on a meta-analysis, the long-term oncological prognosis of patients with PDAC is significantly associated with specific categories of TILs, specifically CD8^+^ T cells [57]. At the present moment, two ongoing clinical trials are currently in the process of recruiting participants. These trials will aim to implement TIL therapy on individuals who are affected by metastatic PDAC (NCT03935893 and NCT01174121). The former trial will assess the efficacy of the adoptive transfer of autologous TILs in combination with fludarabine and cyclophosphamide, while the latter trial will investigate the efficacy of young TILs in combination with aldesleukin (a recombinant analog of IL-2), pembrolizumab, cyclophosphamide, and fludarabine. To further explain, the young-TIL approach involves minimal in vitro culturing of TILs and does not select for tumor recognition before they are rapidly expanded and infused into the patients. This method has achieved objective response rates similar to those of used TILs screened for tumor recognition, without introducing any additional toxicities [268].

#### Genetically modified T cells therapy

##### TCR-engineered T-cell therapy

The production of TCR-engineered T cells involves modifying T cells outside the body to express TCRs that recognize tumor antigens. TCRs have the capacity to detect peptides displayed by both MHC class I and II [269]. Investigating the safety and effectiveness of autologous MSLN-specific TCR T cells in patients with stage IV pancreatic cancer is the objective of a phase I clinical trial (NCT04809766). In this trial, autologous MSLN-specific TCR-T Cells were used in combination with bendamustine, cyclophosphamide, and fludarabine. Patients received three infusions of TCR-TMSLN cells every 21 days following leukapheresis. The main focus was on safety and dose-limiting toxicities, but the study also looked at ORR, PFS, and OS. The goal is to achieve a significant ORR of 20% among the 15 participants [270].

The patient with metastatic PDAC received autologous TCR-engineered T cells as treatment. These modified T cells express two allogeneic human leukocyte antigen (HLA)-C*08:02-restricted KRAS G12D in a clonal manner. Remarkably, the patient's visceral metastasis showed regression, with an overall partial response of 72%. Furthermore, the therapeutic effect persisted for a duration of 6 months. Moreover, after six months of the T-cell transfer, the modified T cells accounted for more than 2% of all circulating T cells in the peripheral circulation [271].

##### *CAR* T-cell therapy

CAR T cells can be compared to the administration of a living drug to patients. At, the CAR T-cell therapies that are accessible are tailored according to each patient's needs. These therapies are created by gathering T cells from the patient and modifying them in the lab to generate CARs on the cell surface. The specific CARs possess the ability to detect and attach themselves to particular proteins, known as tumor antigens, located on the outer surface of cancer cells. Despite its impressive clinical outcomes in the treatment of specific subgroups of B-cell leukemia or lymphoma, CAR T-cell therapy encounters numerous impediments that impede its widespread application in the treatment of solid tumors and hematological malignancies. Impediments such as life-threatening toxicities, cytokine release syndrome (CRS), inadequate anti-tumor efficacy, antigen escape, and limited trafficking all pose obstacles to the successful implementation of CAR T-cell treatment [272, 273]. Tables 2 and 3 provide a comprehensive overview of data regarding CAR T-cell therapy in both preclinical and clinical trial settings.

**Table 2:** Evidence from clinical studies supporting the use of CAR T-cell therapy for pancreatic cancer treatment

Target	CAR’s molecular structure	Combination therapy	Clinical outcomes (= number of patients)	References
CD133	Anti-CD133 ScFv + Human CD137 + CD3ζ	Nab-paclitaxel + Cyclophosphamide	PR = 2, SD = 3, PD = 2	[307]/NCT02541370
EGFR	Anti-EGFR ScFv + CD8α + CD137 + CD3ζ	Nab-paclitaxel + Cyclophosphamide	PR = 4, SD = 8, PD = 2	[290]/NCT01869166
HER2	Anti-HER2 ScFv + CD8α + CD137 + CD3ζ	Nab-paclitaxel + Cyclophosphamide	PR = 0, SD = 0, PD = 2	[286]/NCT01935843
MSLN	Anti-MSLN ScFv + 4- 1BB + CD3ζ	N/A	SD = 2, PD = 1	[301]/NCT01897415

EGFR: Epidermal growth factor receptor, HER2: Human epidermal growth factor receptor 2, MSLN: Mesothelin, N/A: Not applicable, ScFv: Single-chain variable fragment, PR: Partial response, SD: Stable disease, PD: Progressive disease

**Table 3:** Clinical trials in pancreatic cancer investigating the effectiveness of CAR T-cell therapy

Target	Agent/intervention	Combination therapy	Participants with pancreatic cancer	Phase	Outcomes*	Status	Reference/NCT Identifier
ROR2	CCT301-59	N/A	18	I	Safety, efficacy, and kinetics of CCT301-59	Unknown	NCT03960060
HER2	CCT303-406	N/A	15	I	Safety, tolerability, DLTs, and MTD	Recruiting	NCT04511871
CD22	CAR-T/CAR-TILs cells contains anti-CD22 CAR and a ScFv fragment of anti-PD-L1	N/A	30	I	ORR, PFS, OS, and AEs	Recruiting	NCT04556669
CD70	Anti-hCD70 CAR transduced peripheral blood lymphocytes	Cyclophosphamide + Fludarabine + Aldesleukin	124	I, II	Safety and regression of CD70 expressing tumors	Recruiting	NCT02830724
CEA	Anti-CEA CAR-T cells	N/A	5	I	No on-target/off-tumor serious AEs above grade 3 Complete metabolic response within the liver Normalization of serum tumor markers and an abundance of CAR^+^ cells in tumor specimens	Completed	[570]/NCT02850536
Claudin18.2	CT041	N/A	110	I, II	No DLTs, treatment-related deaths, and severe CRS One patient received tocilizumab 2 patients had partial response, 2 had stable disease, and 3 had progression of disease	Active, not recruiting	[571]/NCT04404595
		Anti-PD-1 + Paclitaxel or Irinotecan or Apatinib	192	I, II	Two patients received the therapy. First patient and second patient had partial and complete response, respectively	Recruting	[312]/NCT04581473
EpCAM	Anti-EpCAM CAR T cells	N/A	60	I, II	Determining toxicity profile, persistence of CAR T cells, and efficacy	Unknown	NCT03013712
EpCAM/TM4SF1	TM4SF1 and EpCAM-positive CAR T-cell therapy	N/A	72	N/A	Determining safety, persistence of CAR T cells, and ORR	Unknown	NCT04151186
MSLN	huCART-meso cells	N/A	18	I	Determining AEs, ORR, PFS, and OS	Recruiting	NCT03323944

^*^In cases where the outcomes of the clinical trial have not yet been released, the goals (primary/secondary outcome measures) of the study are mentioned

AEs: Adverse events, BCMA: B-cell maturation antigen, CAR: Chimeric antigen receptor, CD: Cluster of differentiation 19, CEA: Carcinoembryonic antigen, CRS: Cytokine release syndrome, DLTs: Dose-limiting toxicities, EGFR: Epidermal growth factor receptor, EpCAM: Epithelial cell adhesion molecule, GD2: Disialoganglioside, GP3: Glypican 3, HER2: Human epidermal growth factor receptor 2, MSLN: Mesothelin, MTD: Maximum tolerated dose, MUC-1: Mucin-1, N/A: Not applicable, ORR: Overall response rate, OS: Overall survival, PSCA: Prostate stem cell antigen, PD-1: Programmed cell death protein 1, PD-L1: Programmed death-ligand 1, PFS: Progression-free survival, ROR2: Receptor tyrosine kinase-like orphan receptor 2, TM4SF1: Transmembrane 4 L Six Family Member 1

**Fig. 4:**
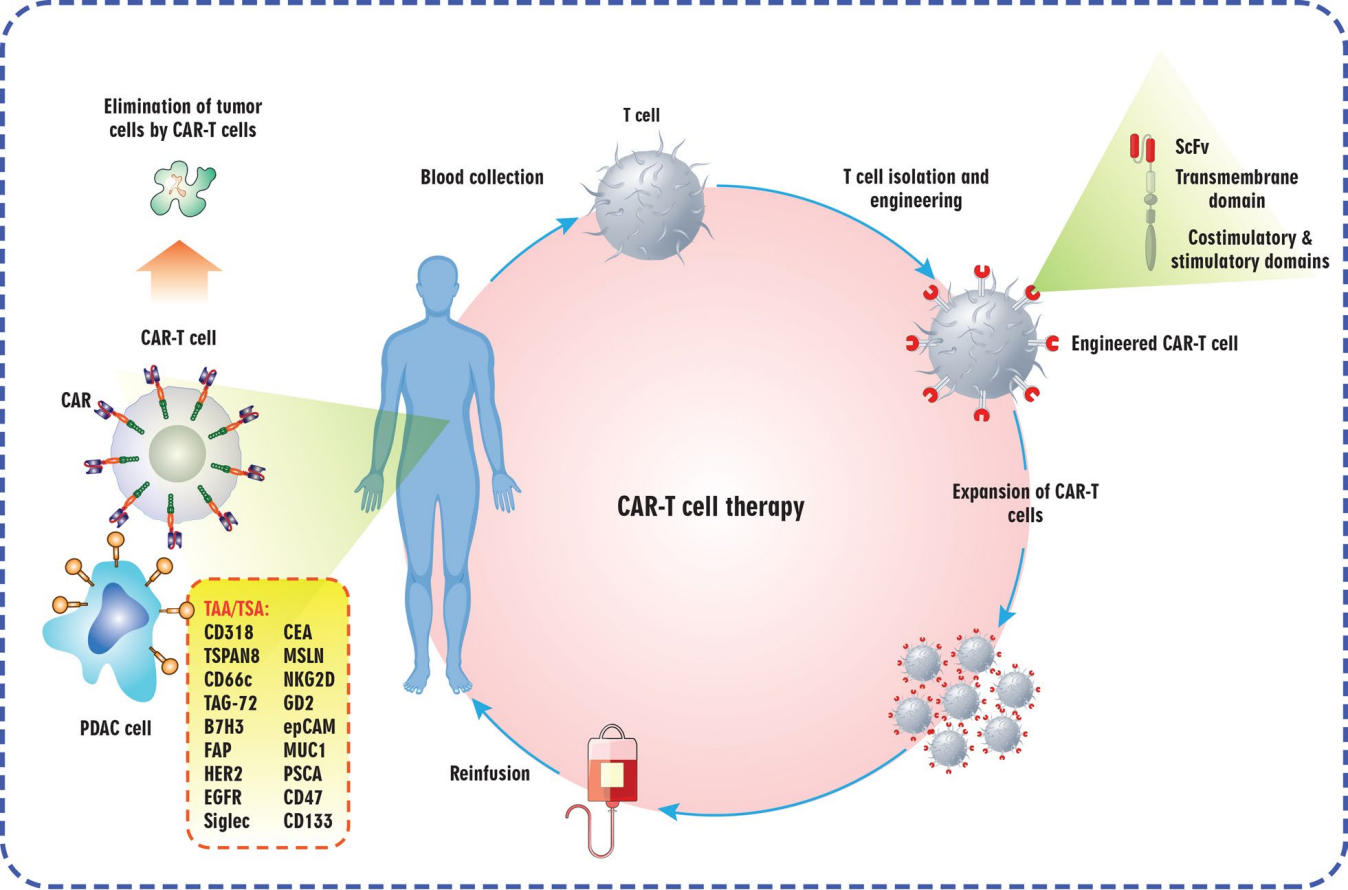
An overview of chimeric antigen receptor (CAR) T cell therapy concept. CAR T cell therapy is a treatment approach, whereby T cells from an individual are modified in a laboratory setting to possess the ability to identify specific antigens found on cancer cells, leading to their elimination. (1) This process involves removing autologous T cells from the patient's blood. (2) Subsequently, the T cells are manipulated by introducing a gene encoding a specialized receptor, known as a CAR, into their genetic makeup through viral vectors. (3) This genetic alteration results in the expression of the CAR protein on the surface of the patient's T cells, thereby creating CAR T cells. These CAR T cells are then multiplied and expanded in laboratory conditions, producing millions of them. (4) Eventually, these CAR T cells are administered to the patient through intravenous infusion. (5) The CAR T cells attach themselves to the cancer cells by binding to the antigens present on their surface and proceed to eradicate the cancer cells. EGFR: Epidermal growth factor receptor; FAP: Fibroblast activation protein; MSLN: Mesothelin; PDAC: Pancreatic ductal adenocarcinoma; ScFv: Single-chain variable fragment; TAA: Tumor-associated antigen; TSA: Tumor-specific antigen

A crucial obstacle to the effective use of cellular immunotherapy for treating PDAC, specifically CAR T-cell therapy, is the lack of suitable tumor-specific antigens. In their research, Schäfer et al. pinpointed CD318, TSPAN8, and CD66c as potential target molecules for CAR T-cell-based immunotherapy in PDAC, among a pool of 371 antigens [274]. Highlighted in the subsequent text are the appropriate therapeutic targets for the CAR T-cell therapy of pancreatic cancer (Fig. 4).

*B7H3 (CD276)* B7H3, a molecule found on the surface of cells, acts as an immune checkpoint and hinders the activation of T-cells and the ability of NK cells to kill. The promise of targeting B7H3 for CAR T-cell therapy arises from its high expression in numerous cancer types while being minimally expressed in healthy tissues [275]. Survival was achieved in mice following treatment with B7H3 CAR T cells, and there were no observed AEs [276]. The outcome of studies conducted in vitro revealed that these cells exhibited a potent ability to suppress the growth of cancer cells in the pancreas [276, 277].

*Fibroblast activation protein (FAP)* FAP is a type-II transmembrane serine protease expressed almost exclusively on CAFs. In mouse models of solid tumors, the growth of tumors can be effectively suppressed by FAP-expressing stromal cells being targeted by CAR T cells designed specifically for FAP [278, 279]. When FAP-specific CAR T cells are administered along with anti-PD-1 treatment, the combination leads to a synergistic reduction in pancreatic tumor growth and significantly elongated survival in mouse models compared to alternative treatment combinations [54].

*Human epidermal growth factor receptor 2 (HER2)* HER2, a glycoprotein located on the cell membrane, performs a function in promoting cell division and distinction during various stages, including embryonic and adult periods. HER2 contributes to tumor progression, growth, and spread by obstructing cell death, triggering the formation of new blood vessels, and boosting cell movement [275]. The expression of the HER2 in pancreatic cancer is controversial [280]. However, it has been detected in 20–60% of PDACs according to certain research studies [281, 282]. Also, HER2 might be a potential target in immunotherapy for a small subset of patients with pancreatic cancer, since a report explains nearly 50% of PDAC cases have a total HER2 expression of 2 + or above [283, 284].

The combination treatment of oncolytic adeno-immunotherapy and HER2-specific CAR-T cells shows promising results in eradicating metastatic PDAC. This combinational therapy enhances the migration of CAR-T cells to the tumor site, while also stimulating systemic host immune responses that improve the overall anti-tumor activity [285]. The clinical effect of anti-HER2 CAR T cells was assessed in a study involving 11 patients, two of whom had metastatic pancreatic cancer. The optimal overall outcome for both patients was disease stability, with a PFS of 5.3 and 8.3 months, respectively [286]. The potential clinical outcomes of the treatment should be proven in clinical trials with a larger sample size including patients with PDAC.

All in all, in different studies, the expression of HER2 in pancreatic cancer is controversial and varies from high-level expression [287] to low-level expression [288], making it a potential target for personalized immunotherapy of PDAC. Thus, it is reasonable that HER2 should not be ignored in such a heterogeneous disease with limited treatment options.

*Epidermal growth factor receptor (EGFR)* The EGFR protein, which spans across the membrane, has the capability of binding to various proteins from the EGF family that are located outside the cell. Around 90% of patients diagnosed with PDAC exhibit an identifiable amount of EGFR [289]. For individuals diagnosed with metastatic pancreatic cancer, the safety and effectiveness of the treatment were demonstrated by a median overall survival (mOS) of 4.9 months among the entire group of 14 patients who received anti-EGFR CAR T cells [290].

*Sialic acid-binding immunoglobulin-type lectin (Siglec)* Cell-surface proteins known as Siglecs exhibit the ability to attach themselves to sialic acid. These proteins are predominantly present in immune cells, belonging to a specific group within the I-type lectins. Targeting sialic acids on tumor cells can be accomplished through direct means as well. A new advancement comprises the development of CAR T cells based on Siglec-7/9, which specifically target tumor cells that express sialic acid, causing a delay in the growth of tumors within a melanoma model [291]. According to a study, Siglec-7 and Siglec-9 ligands are specifically expressed by PDAC cells, indicating the potential effectiveness of CAR T cells in combating PDAC [292]. Furthermore, enhancing the effectiveness of solid tumor cellular immunotherapy is greatly facilitated by the cancer cell desialylation approach that reverses the state of immune evasion. By eliminating the Siglec-5 and Siglec-10 genes, it became possible to make a CAR macrophage that exhibits enhanced anti-cancer activity as a result of blocking the glycoimmune checkpoint [293].

*Carcinoembryonic antigen (CEA)* In order to evaluate the efficacy of CEA-specific CAR T cells in combination with recombinant human IL-12 for the treatment of various solid tumors, an experiment was conducted. The findings illustrated that the incorporation of rhIL-12 alongside anti-CEA CAR T cells notably augmented their capacity to suppress the proliferation of pancreatic tumor cells when compared to solely utilizing CEA CAR T-cell treatment [294]. Regarding the central role of IL-12 in CAR T cells, a study has proven that membrane-bound IL-12 in CAR T cells targeting TAG72 promotes anti-tumor responses against human ovarian cancer xenograft models [295]. However, there is a need to apply this approach in PDAC that has not been met.

Additionally, CEACAM7 (CGM2), which is a part of the CEA protein family, may serve as a potential target for PDAC and is specifically present exclusively in the colon and the pancreas. The remission of xenograft tumors occurs as a result of the targeted destruction of pancreatic cancer cells expressing the specific antigen by CAR T cells designed to recognize CEACAM7 [296].

*Mesothelin (MSLN)* CAR T cells have the ability to be altered in a way that enables them to identify a cell surface antigen called MSLN. This antigen is associated with the invasion of tumors and is present in mesothelial tissues, albeit in small amounts. However, it is highly expressed in PDAC [275]. A potent anti-MSLN hYP218 CAR T cells possess improved abilities to infiltrate and remain in tumors, enhancing their effectiveness in combating pancreatic cancer in vitro and in vivo [297]. CAR T cell therapy, which targets both MSLN and CD19 simultaneously, proved to be a safe and well-tolerated approach in treating individuals suffering from metastatic PDAC [298]. In orthotopic animal models of human pancreatic cancer, it was demonstrated that MSLN-specific CAR T cells are efficient [299]. Mice with extremely aggressive PDAC experience tumor shrinkage when subjected to a mixture of MSLN-redirected CAR T cells and TNF-α/IL-2-armed oncolytic adenoviruses [300]. In a phase 1 trial, T cells engineered to express a CAR specific for MSLN were tested in six patients with chemotherapy-refractory metastatic PDAC. The treatment was well tolerated, with no serious toxicities. Disease stabilized in two patients, and one patient showed a significant reduction in tumor metabolic activity, providing evidence of the potential anti-tumor activity of these engineered T cells [301].

*Disialoganglioside (GD2)* GD2, present on the external cellular membrane, is integral to the immunological characteristics of mammalian cells; however, it rarely elicits an immune reaction. Due to the prevalence of GD2 in embryonal malignancies such as brain tumors and its infrequent manifestation in healthy cells, it is viable to target GD2 molecules using CAR T cells specific to this molecule [275].

*Natural killer group 2D (NKG2D)* The NKG2D receptor shows potential as a target for immunotherapy of malignant neoplasms. CAR T cells specific to NKG2D have been employed in the treatment of patients with hematologic and solid tumors. An evaluation was conducted by researchers to determine the practicality and safety of NKG2D-specific CAR T cells, resulting in the discovery that their capacity to multiply and endure within the body was restricted. Gao and colleagues have successfully suppressed the 4.1R gene in NKG2D-specific CAR T cells, thereby augmenting the efficacy of CAR T cells in combatting pancreatic carcinoma [302].

*Epithelial cell adhesion molecule (EpCAM)* EpCAM is a transmembrane glycoprotein of type I that is excessively expressed in various carcinomas, for instance, colon, stomach, PDAC, and endometrial malignancies. Its connection to the Wnt/β-catenin signaling pathway has been observed, which is believed to trigger inadequate infiltration of T-cells in different human malignancies [275]. A number of clinical trials have been registered to utilize EpCAM-specific CAR T cells in individuals suffering from pancreatic cancer (NCT04151186 and NCT03013712). In these trials, outcomes like toxicity profile, survival time and persistence of CAR T cells in vivo, and anti-tumor efficacy will be measured.

*Mucin-1 (MUC-1; CD227)* At the apical surface of epithelial cells, the transmembrane mucin glycoprotein MUC-1 shows a high level of expression. In more than 80% of human PDAC, MUC-1 is excessively expressed. This excessive expression of MUC-1 is associated with a grim prognosis and increased metastasis. Moreover, through the upregulation of multidrug resistance genes, MUC-1 boosts chemo-resistance in pancreatic cancer cells [303]. In xenograft models of pancreatic cancer, there was successful inhibition of tumor growth by anti-MUC-1 CAR T cells, which exhibited effective targeting capabilities and induced cytotoxicity [304]. To investigate the effectiveness of MUC-1-specific CAR T cells on individuals suffering from pancreatic cancer, two clinical trials have been officially registered under the codes NCT05239143 and NCT04025216. The former clinical trials demonstrated three patients with different types of cancer have been treated with anti-MUC-1 CAR T cells, showing good tolerance and no observed toxicities [305]. Latter clinical trial showed safety and preliminary efficacy in treating various solid tumors, with no dose-limiting toxicities observed in the six treated patients and preliminary results indicating stable disease in all patients [306].

*CD133* Both hematopoietic cells and epithelial cells exhibit the pentaspan transmembrane glycoprotein CD133. CD133 has been identified not only in pancreatic cancer CSCs but also in different tumors like hepatocellular and gastric carcinomas, emphasizing its widespread presence in malignancies. In 50% of cases involving pancreatic cancer, the expression of CD133 was observed to be significantly high [289]. CD133-specific CAR T cells were administered to 7 individuals who suffer from PDAC during a phase 1 clinical trial. Before the infusion of CAR T cells, the patients were treated with cyclophosphamide and nab-paclitaxel. The outcomes of the trial exhibited 3 instances of disease stabilization, 2 instances of partial remission, and 2 instances of disease progression [307].

*Prostate stem cell antigen (PSCA)* Initially, PSCA was identified as a surface glycoprotein that consists of 123 amino acids and is linked to glycophosphatidylinositol. Its function remained unknown, although it showed significant presence in prostate cancers while exhibiting minimal levels in the prostate epithelium, urinary bladder, kidney, esophagus, stomach, and placenta. Further investigations confirmed its amplified expression in various human cancers, such as pancreatic cancer, while being absent in a healthy pancreas. By employing a humanized mouse model for pancreatic cancer, it was observed that CAR T cells specifically targeting PSCA were able to prompt the eradication of tumors [308].

*CD47* CD47, an immunoglobulin superfamily member, frequently exhibits heightened expression in various hematological and solid cancer tumors. Its crucial function involves the inhibition of phagocytosis, leading to enhanced tumor survival, metastasis, and angiogenesis. CD47 is recognized as a "don’t eat me" since it bonds with signaling regulatory protein alpha (SIRP-α) and obstructs the phagocytosis of cancer cells [309]. The blocking of pancreatic xenograft tumor growth is efficiently accomplished and cancer cells are effectively killed by CD47-specific CAR-T cells [310]. A significant challenge in using CD47-CAR-T cells could be the potential detrimental effects on red blood cells and platelets due to the expression of CD47 on these cells. However, in this study, CD47-CAR-T cells were administered intratumorally, which may prevent the induction of toxic effects on other cells. Therefore, the clearing of blood cells during systemic injection of this treatment should be considered as a potential AE.

*Claudin18.2 (CLDN18.2)* The protein known as CLDN18.2 is an isoform-specific to the stomach of the CLDN18 tight junction protein. This protein is found in high levels in various types of cancer, particularly those affecting the digestive system like pancreatic cancer. Therefore, it could be a promising candidate for cancer treatment strategies [311–313]. Studies indicated a positive response rate for CT041, which are autologous T cells that have been genetically modified to express a CAR targeting CLDN18.2, in cases of digestive system malignancies [311, 314–316]. Two patients with metastatic pancreatic cancer were treated with anti-CLDN18.2 CAR T cell therapy (CT041) after standard treatments failed. Both patients experienced CRS, which was managed with tocilizumab. The first patient showed a partial response with a significant reduction in lung metastasis, while the second patient achieved a complete response. Both cases experienced an increase in CD8^+^ T cells and Treg cells, a decrease in CD4^+^ T cells and B cells, an increase in IL-8, and a decrease in TGF-β1. The tumors were well-controlled at the last follow-up [312].

##### *CAR*-NK cell therapy

CAR NK cell therapy is a promising strategy in cancer treatment that seeks to enhance the cancer-fighting power of NK cells. CAR-NK cells are engineered to express CARs that recognize specific antigens in cancer cells, which allows them to target and kill cancer cells more effectively [106, 317]. Table 4 provides a comprehensive comparison between CAR T cells and CAR NK cells. Compared to CAR T cells, CAR NK cells possess multiple benefits. Their limited lifespan implies a decreased likelihood of unintended harm to healthy cells (referred to as on-target/off-tumor toxicity). The unique set of cytokines they release signifies a reduced potential for CRS and neurotoxicity. Furthermore, their lower propensity for alloreactivity facilitates the production of off-the-shelf allogeneic CAR NK cells derived from NK cell lines [318]. However, they may have several negative points, which restrict their broad application in clinical contexts. First of all, difficulties in proper antigen selection, antigen heterogeneity, donor selection, challenges in designing an effective CAR, and difficulties in producing and storing CAR NK cells are fundamental hurdles in this type of treatment modality [106, 319]. Secondly, issues such as NK cell infiltration into tumor sites and the short half-life of NK cells must be considered [319]. This short lifespan can necessitate repeated administrations to achieve a durable response. Furthermore, the need for continuous immune surveillance and prevention of cancer recurrence requires the reprogramming of CAR NK cells with memory cell properties and long-term survival in vivo [320, 321]. Lastly, NK cells have several inhibitory killer-cell immunoglobulin-like receptors (KIRs) on their surface, which are cognate with their ligands, HLA molecules. Thus, the universally expressed HLA molecules on nucleated cells can inhibit CAR NK cell function [318]. Thus, the translation of CAR NK cell therapy from bench to bedside requires addressing the aforementioned challenges properly.

**Table 4:** A comprehensive comparison between CAR T cell and CAR NK cell therapies

Difference	CAR T cell	CAR NK cell	References
Key markers	TCR, CD3	CD16, CD56	[572]
Receptor activated		NKG2D, NKG2C, NKp44, KIR	[572]
CAR generations	Five generations	Four generations	[106, 573–576]
Intracellular and co-stimulatory signaling domains	CD3ζ, CD28, 4-1BB (CD137), CD27, CD40, OX40 (CD134)	CD3ζ, DAP10, DAP12, 2B4 (CD244), 4-1BB, CD28	[318, 576–578]
Production of memory cells	+ + +	+	[106, 579, 580]
Off-the-shelf products	+ (HLA-matched allogeneic CAR T cells)	+ (non-HLA-matched allogeneic and NK cell lines like NK-92 cells)	[318, 581]
Time for manufacturing	1 to 2 weeks Rapid manufacturing in 24 h is also reported	Exact timeline can vary (typically 2 to 4 weeks)	[582, 583]
Redosing	Not limited by cell number Risk of alloimmunization	Not limited by cell number	[319]
In vitro expansion during manufacturing	+ (Autologous or allogeneic T cells can be expanded after CAR transduction.)	+ (autologous NK cell, iPSCs, and NK-92 cells can be pre-expanded before CAR transduction.)	[318, 584]
In vivo persistence	Relative long-term persistence of functional CAR T cells (armored CAR T cells) Intermediate (weeks to months) In some patients with leukemia, CAR-T cells can be identified several years after being infused	Low and limited persistence in the absence of cytokine Short-term lifespan without IL-15 Cord blood-derived CAR NK cells can persist for at least 12 months (Liu et al.)	[572, 585, 586]
Immune cell sources	Autologous PBMCs PBMCs from well-matched donor	Peripheral blood Umbilical cord blood and cord blood HPSCs Differentiated pluripotent stem cells (e.g., iPSCs) NK-92 cell line (an immortalized NK lymphoma cell line)	[106, 572, 584]
Cytotoxicity mechanisms	In a CAR-dependent manner Perforin and granzyme Inducing apoptotic signaling pathways in tumor cells	In both CAR-dependent and -independent manners Perforin and granzyme ADCC through CD16 Inducing apoptosis	[318, 572]
Risks and toxicities	+ + + (CRS, neurotoxicity, and GVHD) Risk of malignancy after treatment (low risk)	+ (less common) A protocol for freezing and thawing needs to be developed and clinically evaluated for a ready-to-use product	[319, 572, 587, 588]
Infiltration to TME	Poor (particularly in cold tumors)	Usually poor	[318, 589]
Combination therapies	Chemotherapy (like cyclophosphamide) Radiotherapy Immune checkpoint inhibitors (like anti-PD-1) Oncolytic viruses Cancer vaccines Immunomodulatory agents Allogeneic hematopoietic cell transplantation Metabolic inhibitors	Immune checkpoint inhibitors (anti-NKG2A antibody, monalizumab, lirilumab, and so on) Immunomodulatory drugs (lenalidomide) Epigenetic modulators (vorinostat) Oncolytic viruses (adenoviruses) Small-molecule inhibitors (GSK3i)	[106, 590]
Clinical trials	Extensive clinical trials Proven effectiveness (at least 6 FDA-approved CAR T cell therapies)	Limited clinical trials No FDA-approved CAR NK cell therapies yet Clinical efficacy reported in some studies	[106, 318, 576]

ADCC: Antibody-dependent cellular cytotoxicity, CAR: Chimeric antigen receptor, CRS: Cytokine release syndrome, FDA: The U.S. food and Drug Administration, GSK3i: Glycogen synthase kinase-3 inhibitor, GVHD: Graft-versus-host disease, HLA: Human leukocyte antigen, HPSCs: Hematopoietic stem and progenitor cells, iPSCs: Induced pluripotent stem cells, KIR: Killer-cell immunoglobulin-like receptor, NK: Natural killer, PBMC: Peripheral blood mononuclear cell, PD-1: Programmed cell death protein 1, TCR: T cell receptor, TME: Tumor microenvironment

When ROBO1 is targeted, CAR-NK immunotherapy accompanied by radiation therapy proves to be more effective in treating human PDAC in an orthotopic mouse model [322]. A study demonstrates the effectiveness of a novel human NK cell-based immunotherapy targeting PSCA. It found that these cells effectively suppressed PSCA^+^ pancreatic cancer in vitro and in vivo. The therapy showed promising results without causing systemic toxicity [323]. Furthermore, the inhibition of tumor growth and enhancement of survival were observed in a mouse model of pancreatic cancer when utilizing a fusion of CAR-NK cells that targeted MSLN, along with cGAMP, an agonist for STING [324]. There are two clinical trials that have been registered for the implementation of ROBO1 and MUC-1-specific CAR NK cells in the existing clinical scenario of immunotherapeutic methods, specifically for patients diagnosed with pancreatic cancer (NCT03941457 and NCT02839954). Outcome measurements include an examination of the safety profile and ORR.

In the realm of immunotherapy for pancreatic cancer, PSCA has recently gained acclaim as a promising contender. Research findings highlight that CAR-NK cells designed to target PSCA demonstrate notable efficacy in combating advanced PDAC in humans, all while ensuring the absence of any harmful effects at a systemic level [323]. These positive outcomes provide a rigorous rationale for the future progression of clinical trials.

Induced pluripotent stem cells (iPSCs) provide a convenient supply of lymphocytes for immunotherapy. These NK cells, derived from iPSCs, express essential NK-defining markers such as CD56 and CD16. They demonstrate cytotoxicity through cytokine secretion and ADCC, showing potential for cancer treatment [325, 326]. The first-in-class, off-the-shelf iPSC-derived NK cell therapy called FT500 is currently being evaluated in a phase I clinical trial. This trial aims to treat advanced solid tumors, including pancreatic cancer. FT500 is administered both as a monotherapy and in combination with checkpoint inhibitor therapy (nivolumab, pembrolizumab, Atezolizumab), IL-2, cyclophosphamide, and fludarabine (NCT03841110).

##### Cytokine-induced killer (CIK) cell therapy

CIK cells form a diverse group of CD8^+^ T cells that were produced from lymphocytes extracted from human peripheral blood and simply expanded ex vivo through incubation with an anti-CD3 antibody, IFN-γ, and IL-2. Through FasL and perforin, they have the ability to eliminate cancer cells. Depending on the existence of the cell surface molecule CD56, CIK cells are additionally categorized into two primary subsets: T cells that are positive for CD3 and CD56, and T cells that are positive for CD3 but negative for CD56 [327]. Adopting CIK cells and transferring them has proven to be highly effective and safe in cancer treatment, as demonstrated by the increased survival of individuals affected by different types of tumors. When utilized alongside chemotherapy, CIK cell therapy exhibits enhanced efficiency in thwarting cancer relapse and enhancing patients' prognosis [24].

Researchers have investigated the application of CIK cells as a potential second-line treatment for advanced pancreatic cancer, which has yielded encouraging outcomes in both standalone usage and when combined with other therapeutic methods. In a phase II clinical investigation, the inclusion of CIK cells alongside gemcitabine-refractory advanced pancreatic cancer demonstrated a mOS of 6.2 months among patients [328]. In advanced pancreatic cancer, gemcitabine-resistant patients who underwent CIK cell therapy in combination with S-1, an oral fluoropyrimidine derivative, demonstrated a mOS of 6.6 months, surpassing the mOS of patients solely treated with S-1 alone (6.1 months) [329]. Following CIK cell therapy, individuals diagnosed with advanced pancreatic cancer exhibit notable enhancements in the OS [330].

### Immune checkpoint-oriented immunotherapy

Immunotherapy has become a leading pillar of cancer treatment, thanks to the triumph of an effective ICB method, mainly exemplified by the approval of ipilimumab in 2011. By inhibiting specific inhibitory immune checkpoints like CTLA-4, PD-1, and PD-L1, ICB actively halts or reverses the development of acquired peripheral tolerance to cancer antigens, consequently restoring T-cell activation [331]. Table 5 presents a comprehensive overview of clinical trials investigating the potential of ICIs and immunomodulatory agents in the treatment of pancreatic cancer. In the subsequent discourse, we elucidate the pivotal significance of immune checkpoints in the therapeutic intervention of pancreatic cancer.

**Table 5:** Clinical trials in pancreatic cancer investigating the effectiveness of immune checkpoint inhibitors

Agent	Target	Combination therapy	Participants	Phase	Outcomes*	Status	NCT identifier/References
Atezolizumab + Selicrelumab (CD40 agonist) + Bevacizumab + Tiragolumab + Tocilizumab	PD-L1, CD40, VEGF, TIGIT, IL-6R	Nab-Paclitaxel + Gemcitabine + Oxaliplatin + Leucovorin + Cobimetinib + Fluorouracil + PEGPH20 + BL-8040 (CXCR4 antagonist) + RO6874281 + AB928 + LSTA1	340	I, II	ORR = 6.1% (n = 66) in atezolizumab plus PEGPH20 group (arm A) and ORR = 2.4% (n = 42) in chemotherapy group (arm B) 65.2% (arm A) and 61.9% (arm B) had grade 3/4 AEs 4.5% (arm A) and 2.4% (arm B) had grade 5 AEs Limited clinical activity	Active, not recruiting	NCT03193190/[591]
Pembrolizumab	PD-1	Onivyde (chemotherapy) + BL-8040 (Motixafortide)	80	II	BL-8040 increased CD8^+^ effector T cells and decreased MDSCs and circulating regulatory T cells DCR = 34.5% (nine patients with stable disease and one patient with partial response) and mOS = 3.3 months (n = 29) in cohort 1 ORR = 32%, DCR = 77%, and mDOR = 7.8 months in cohort 2 (n = 22)	Completed	NCT02826486/[506]
		Epacadostat (INCB24360) + CRS207 + GVAX cancer vaccine	40	II	Determining the recommended dose of epacadostat Assessing survival	Active, not recruiting	NCT03006302
		Cyclophosphamide + GVAX cancer vaccine + Radiotherapy	58	II	mOS = 15.8 months and distant metastasis free survival = 9.7 months Grade 3 AEs included 1 case each of dermatitis, colitis, DKA, nephritis, and pneumonitis Increased densities of granzyme B^+^CD8^+^ T cells, TH1, and TH17 in TME Increased M2-like tumor-associated macrophages in TME	Completed	NCT02648282/[592, 593]
		Olaptesed pegol (NOX-A12)	20	I, II	25% of patients achieved stable disease OS = 42% at 6 months and 19% at 12 months mPFS = 1.87 months No objective responses NOX-A12 monotherapy stimulated TH1 cytokines (IFN-γ and IL-2)	Completed	NCT03168139/[594]
		Defactinib	59	I, II	Determining safety and tolerability Determining early indications of improved anti-cancer immunotherapy	Recruiting	NCT02758587
		Acalabrutinib (ACP-196)	77	II	14.3% (monotherapy arm; n = 37) and 15.8% (combination therapy arm; n = 40) had grade 3–4 AEs ORR = 0% and DCR = 14.3% in monotherapy arm Vs. ORR = 7.9% and DCR = 21.1% in combination therapy arm mPFS = 1.4 months	Completed	NCT02362048/[595]
		Gemcitabine hydrochloride + Nab-paclitaxel + Paricalcitol	9	I	Determining the safety Determining number of tumor infiltrating lymphocytes in resected pancreatic cancer subjects Evaluating OS, DFS, PD-L1 expression, CD8^+^CD45RO^+^ cells, and immune signature in TME	Completed	NCT02930902
		Defactinib + Gemcitabine	43	I	DCR = 58.8% (n = 17) with one partial response and nine stable disease mPFS = 4.2 months and OS = 9.1 months	Completed	NCT02546531/[596]
Cabiralizumab (FPA008) + Nivolumab (BMS-936558)	CSF-1R, PD-1	N/A	313	I	Determining mPFS, OS, ORR, mDOR, pharmacokinetics, and safety	Completed	NCT02526017
AMG820 + Pembrolizumab	CSF-1R, PD-1	N/A	117	I, II	87.9% with grade ≥ 3 AEs Most common AEs: increased aspartate aminotransferase (59.5%), fatigue (48.3%), periorbital/face edema (48.3%), and rash/maculopapular rash (37.1%) Immune-related partial response in 3 patients (3%; DOR 9.2, 10.0, 12.5 months) Immune-related stable disease in 39 patients (34%)	Completed	NCT02713529/[597]
Pembrolizumab + IMC-CS4	PD-1 + CSF-1R	Cyclophosphamide + GVAX cancer vaccine	12	I	Grade 3/4 AEs: rash and diarrhea mDFS/mOS = 12.6/20.4 month and pathologic response rate = 78% 75% of patients (6/8) had a > 80% increase of CD8^+^ T cells	Completed	NCT03153410/[415]
Pembrolizumab	PD-1	ARRY-382 (PF-07265804; CSF-1R inhibitor)	82	I, II	Limited clinical benefits AEs: increased transaminases and creatine phosphokinase 3.7% of patients (1/27) had a partial response lasting 2.4 months (n = 27)	Terminated due to insufficient efficacy	NCT02880371/[598]
Durvalumab (MEDI4736)	PD-L1	Pexidartinib (PLX3397; a CSF-1R-directed tyrosine kinase inhibitor)	48	I	No unexpected toxicities 15% patients had stable disease (n = 47) Increased CSF-1 and FLT3-L concentrations and decreased CD14^low^CD16^high^ monocytes and DCs in blood Impaired IFN-λ/IL-29 production by type 1 conventional DCs Limited antitumor clinical activity	Completed	NCT02777710/[599]
Ipilimumab	CTLA-4	Gemcitabine hydrochloride	21	I	Common grade 3 or 4 AEs: anemia (48%), leukopenia (48%), and neutropenia (43%) ORR = 14% (3/21) and seven patients had stable disease mOS = 6.90 months and mPFS = 2.78 months Median response duration = 11 months	Completed	NCT01473940/[600]
Botensilimab (AGEN1181)	CTLA-4	Gemcitabine + Nab-paclitaxel	78	II	Determining PFS, complete response, ORR, DOR, change from baseline in carbohydrate antigen 19–9, and potential AEs	Recruiting	NCT05630183
Nivolumab	PD-1	Nab-paclitaxel + Gemcitabine + Carboplatin	114	I	Dose-limiting toxicity = One hepatitis among 6 patients 96% (48/50) had grade 3/4 AEs (n = 50) 2% (1/50) had grade 5 AEs mPFS = 5.5 months, mOS = 9.9 months, and ORR = 18% mPFS/mOS = 5.5/9.7 months (PD-L1 < 5%) and 6.8/11.6 months (PD-L1 ≥ 5%) Significant increased Ki67^+^ CD8^+^/CD4^+^ cells	Completed	NCT02309177/[601]
		Cyclophosphamide + GVAX cancer vaccine + Radiotherapy	30	II	Progressive disease = 5.5% (1/18), occult intra-operative metastatic disease = 16.6% (3/18) and definitive surgical resection = 77.7% (14/18; n = 18) mOS = 20.4 months (median follow-up of 20.5 months) and pathologic response rate = 35% Grade ≥ 3 AEs: two cases (nivolumab-related autoimmune hepatitis and SBRT-related pancytopenia)	Completed	NCT03161379/[602]
		Nab-Paclitaxel + Gemcitabine + APX005M (sotigalimab; CD40 agonist)	129	I, II	Arm A (nivo/chemo; n = 34): 1-year OS = 57.7% (*P* = 0.006), mOS = 16.7 months, mPFS = 6.4 months, ORR = 50%, DCR = 74%, and mDOR = 7.4 months Arm B (sotiga/chemo; n = 36): 1-year OS = 48.1% (*P* = 0.062), mOS = 11.4 months, mPFS = 7.3 months, ORR = 33%, DCR = 78%, and mDOR = 5.6 months Arm C (sotiga/nivo/chemo; n = 35): 1-year OS = 41.3% (*P* = 0.233), mOS = 10.1 months, mPFS = 6.7 months, ORR = 31%, DCR = 69%, and mDOR = 7.9 months Common AEs: CRS (0%, arm A; 24%, arm B; 34%, arm C), thrombocytopenia (50%, arm A; 57%, arm B; 63%, arm C), elevated liver function tests (67%, arm A; 81%, arm B; 74%, arm C)	Completed	NCT03214250/[603]
		CRS-207 vaccine + GVAX + Cyclophosphamide	93	II	mOS = 5.9 months in nivolumab group (n = 51; arm A) and 6.1 months in group without nivolumab (n = 42; arm B) ORRs = 4% in arm A and 2% in arm B grade ≥ 3 AEs = 35.3% in arm A and 11.9% in arm B Changes in TME include an increase in CD8^+^ T cells and a decrease in CD68^+^ myeloid cells	Completed	NCT02243371/[604]
Cabiralizumab + Nivolumab	CSF-1R + PD-1	Nab-paclitaxel + Onivyde + Fluorouracil + Gemcitabine + Oxaliplatin + Leucovorin + Irinotecan hydrochloride	202	II	Determining PFS, ORR, DOR, OS, and AEs	Completed	NCT03336216
Durvalumab + Tremelimumab	PD-L1, CTLA-4	Radiotherapy	65	I, II	No dose limiting toxicities Grade 3–4 AEs: lymphopenia and anemia 3 patients with partial response and 7 patients with stable disease (n = 31) mPFS/OS = 1.7/3.4 months (cohort 1), mPFS/OS = 2.6/9.1 months (cohort 2), mPFS/OS = 1.6/3 months (cohort 3), and mPFS/OS = 3.2/6.4 months (cohort 4)	Completed	NCT02311361/[605]
Nivolumab + Ipilimumab	PD-1, CTLA-4	Radiotherapy	30	II	ORR = 4% (1/28), DCR = 11% (3/28), PFS = 2.3 months, and OS = 2.9 months 1 patient had a complete response at 13 months Grade 3–4 AEs: lymphopenia (grade 4 in 1 patient), neutropenia, fatigue, muscle weakness, ALT/AST increase, hepatobiliary dysfunction, acute kidney injury, hyperglycemia, and hypokalemia	Active, not recruiting	NCT04361162/[606]
Anti-PD-1 monoclonal antibody	PD-1	Radiotherapy	21	II	Determining ORR, OS, and AEs	Unknown	NCT03374293
Nivolumab (BMS-936558) + Urelumab (BMS-663513) + BMS-986253	PD-1, CD137 (4-1BB), IL-8	Cyclophosphamide + GVAX cancer vaccine	76	II	Arm A (GVAX/cyclopho; n = 16): mDFS/mOS = 13.90/23.59 months Arm B (nivo/GVAX/cyclopho; n = 14): mDFS/mOS = 14.98/27.01 months Arm C (urelu/nivo/GVAX/cyclopho; n = 10): mDFS/mOS = 33.51/35.55 months Increased intratumoral CD8^+^CD137^+^ cells in arm C compared t arm B (*P* = 0.003) Improved DFS (*P* = 0.242 and *P* = 0.173) and OS (*P* = 0.377 and *P* = 0.279) in arm C compared to arms A and B	Recruiting	NCT02451982/[381]
BGB-A425 + Tislelizumab (BGB-A317) + LBL-007	TIM-3, PD-1, LAG-3	N/A	358	I, II	Determining AEs, maximum tolerated dose, ORR, DOR, DCR, PFS, and pharmacokinetics	Recruiting	NCT03744468

^*^In cases where the outcomes of the clinical trial have not yet been released, the goals (primary/secondary outcome measures) of the study are mentioned

AEs: Adverse events, CSF-1R: Colony stimulating factor 1 receptor, CTLA-4: Cytotoxic T lymphocyte antigen-4, DC: Dendritic cell, DCR: Disease control rate, ICB: Immune checkpoint blockade, IL-6R: Interleukin 6 receptor, LAG-3: Lymphocyte activation gene-3, mDFS: Median disease-free survival, mDOR: Median duration of response, mOS: Median overall survival, N/A: Not applicable, ORR: Objective response rate, PD-1: Programmed cell death protein 1, PD-L1: Programmed death-ligand 1, mPFS: Median progression-free survival, TH1: Type 1 T helper, TIGIT: T-cell immunoreceptor with Ig and ITIM domains, TIM-3: T-cell immunoglobulin and mucin domain-containing protein 3, TME: Tumor microenvironment, VEGF: Vascular endothelial growth factor

#### Inhibitory immune checkpoints

##### PD-1/PD-L1 *axis*

The PD-1/PD-L1 axis has been studied in relation to immune checkpoint molecules in pancreatic cancer following the successful use of anti-PD-1/PD-L1 treatment in melanoma. PD-1 is a member of the B7-CD28 protein family and its expression is associated with T-cell exhaustion. PD-1 ligands (PD-L1 and PD-L2) are expressed by tumor cells, MDSCs, TAMs, and tumor-infiltrating DCs. Engagement between PD-1 and PD-L1 leads to T-cell exhaustion by blocking T-cell activation [332, 333]. Certain malignancies have demonstrated promising results when treated solely with PD-1/PD-L1 inhibitors [334, 335]. PD-L1 inhibitors elicit different reactions in individuals, as evidenced by some PD-L1 positive patients exhibiting unfavorable responses while some PD-L1 negative patients responding favorably. This implies the potential involvement of other PD-1 ligands, like PD-L2, in impacting the efficacy of PD-1 axis immunotherapy in specific cancers. A body of research accentuates the notion that PD-L2 influences the anti-PD-1 axis immunotherapy, particularly in PDAC [336, 337]. Chemotherapy-induced senescent cancer cells modify the TME, promoting immunosuppression and pancreatic tumor growth. PD-L2 is highly upregulated in senescent cancer cells, helping them evade the immune system and persist within tumors. Blocking PD-L2 in combination with chemotherapy leads to tumor regression and remission in mice [338], offering a promising therapeutic strategy targeting senescence-induced vulnerabilities.

Combination immunotherapy targeting PD-L1 and CCL5 has shown benefits in PDAC by decreasing Treg and TAM infiltration, inducing CD8^+^ T-cell activation, promoting tumor regression, and improving OS [339]. Tumor regression, improved OS, and the generation of anti-tumor memory cells were achieved by the joint action of anti-tumor necrosis factor receptor 2 (TNFR2) and PD-L1 monoclonal antibodies, by reducing the infiltration of Tregs and TAMs while activating CD8^+^ T-cells in PDAC microenvironment [340]. ADH-503, an agonist of CD11b, exerts an agonistic influence on innate immune responses, leading to a reprogramming effect. This reprogramming enhances the response of innate immune responses towards immunotherapies, specifically anti-PD-L1 antagonists and anti-4-1BB agonists, thereby facilitating a more effective therapeutic outcome in the treatment of pancreatic cancer [341]. A bispecific immunocytokine (PD-1/IL-2 complex) targeting of PD-1 and IL-2Rβγ enhances tumor-antigen-specific T-cell activation while reducing Treg-mediated suppression. The use of this immunocytokine, combined with radiotherapy, attenuates the progression of pancreatic cancer and impedes its metastatic potential [342].

##### CTLA-4 (CD152)

CTLA-4 is predominantly found in Tregs and its expression increases when T-cells are activated. CTLA-4 works intrinsically by suppressing the co-stimulatory signal within the cell, inhibiting T-cell activation. It also acts externally by removing CD80 and CD86 from APCs, which reduces the response of effector T cells [332]. Controlling the pathway of CTLA-4/CD80 regulates the entry of T cells into the microenvironment of pancreatic cancer. By interrupting the interaction between CTLA-4 and CD80, one can induce the infiltration of CD4^+^ and CD8^+^ T-cells into the microenvironment of PDAC [343]. Pancreatic tumors can be regressed by inhibiting both IL-6 and CTLA-4, and this regression occurs through a T cell and CXCR3-dependent mechanism [344].

##### LAG-3

Cancer cells utilize the LAG-3 signaling pathway to escape the immune system's detection. Through interaction with Galectin-3, activated T cells experience decreased functionality. Moreover, the activity of plasmacytoid DCs, responsible for initiating the growth of naïve T cells, is hindered by LAG-3. Additionally, LAG-3 has the capacity to regulate T-cell proliferation, reduce memory and effector T-cell immune responses, and heighten immunosuppression through the suppression mediated by Tregs [332]. Pancreatic cancer patients with TILs that express LAG-3 exhibit lower rates of DFS [345]. Anti-tumor immunity and enduring response in pancreatic cancer can be achieved by directing attention towards T cell checkpoints 4-1BB and LAG-3, alongside myeloid cell CXCR1/CXCR2 [346].

##### TIGIT

The expression of TIGIT occurs on the surface of immune cells and results in the inhibition of T-cell stimulation. By attaching to CD155 and CD112, TIGIT generates signals that suppress the activation of T-cells. Additionally, TIGIT can competitively bind to CD226 or CD96 along with CD155 and CD112 in order to suppress the active signal received by T-cells [347]. Increased PD-1 and TIGIT expression were evident in intratumoral T cells [348]. Thus, to optimize the responses of CD8^+^ T cells against tumors, it is necessary to co-block the TIGIT and PD-1 inhibitory pathways due to their mechanistic convergence [349]. The co-blockade of PD-1 and TIGIT on tissue-resident memory T cells in PDAC revitalizes them [350]. In pancreatic cancer, the CD155/TIGIT axis plays a significant role in boosting and sustaining immune evasion [351]. Combining TIGIT and PD-1 blockade enhances the efficacy of vaccinations in a model of pancreatic cancer [352]. The reinvigoration of T lymphocytes specific to pancreatic tumor cells occurred as a result of the co-blockade of TIGIT/PD-1 and the stimulation of CD40 agonist [351]. A research study uncovered that interactions in samples of human PDAC decrease following chemotherapy, specifically between TIGIT on CD8^+^ T cells and its receptor on cancer cells. TIGIT was identified as the primary inhibitory checkpoint molecule of CD8^+^ T cells, revealing that chemotherapy greatly affects the PDAC TME and potentially enhances resistance to immunotherapy [353].

##### V-domain Ig-containing suppressor of T-cell activation (VISTA)

VISTA is an original member of the B7 family checkpoint molecules. It exerts a distinctive influence on cancer immune evasion through its distinct expression patterns and functions. In contrast to checkpoints that mainly control T-cell effector function and exhaustion, VISTA has various roles. It aids in the functioning of MDSCs, governs the activation of NK cells, promotes the survival of Tregs, restricts antigen presentation on APCs, and also maintains T cells in a state of rest [354–356]. The expression of VISTA is associated with a more favorable prognosis in cases of pancreatic cancer [357]. Pancreatic cancer exhibits an increased expression of the immunological checkpoint VISTA. It has been shown that the activation of VISTA hinders the production of cytokines by T cells that are obtained from metastatic pancreatic cancers [358]. Given this, monoclonal antibodies against VISTA could potentially function as a beneficial immunotherapeutic approach for individuals diagnosed with pancreatic cancer [357, 358].

##### CD39/CD73 *axis*

Extracellular adenosine is a metabolite that suppresses the immune system and affects adversely both innate and adaptive immune responses. It is accumulated through the actions of two ectonucleotidases, CD39 and CD73. Adenosine exerts its immunosuppressive effects by binding to A2A receptors on lymphoid and myeloid cells, as well as A2B receptors on myeloid cells. These A2B receptors are frequently overexpressed in cancer cells and have been found to promote tumor growth, spread, and resistance to chemotherapy [359, 360]. In PDAC, the levels of CD73 are notably elevated compared to other types of cancer. This correlation is associated with negative clinical results [361]. The findings of a study highlight the significant role of CD39 and CD73 in promoting PDAC progression. The expression of these ectonucleotidases was associated with worse survival outcomes in human PDAC samples and disrupted the positive impact of tumor-infiltrating CD8^+^ T cells. Furthermore, targeting both CD39 and CD73 demonstrated superior anti-tumor activity compared to individual inhibition, emphasizing the potential of these molecules as therapeutic targets in PDAC [362]. Several anti-CD73/CD39 antibody-oriented clinical trials are underway, which will assess the effectiveness of agents like oleclumab or MEDI9447 (anti-CD73; NCT02503774), TTX-030 (anti-CD39; NCT03884556), and CPI-006 or mupadolimab (anti-CD73; NCT03454451) [363] alone or in combination with other ICIs. Regarding NCT02503774, the study involved the treatment of 192 patients with oleclumab and durvalumab (anti-PD-L1), with no instances of dose-limiting toxicities during the escalation phase. The most frequently observed side effects were fatigue, diarrhea, and rash. While the escalation phase showed no objective response, the expansion cohorts demonstrated some positive response rates [364].

#### Bispecific antibodies (BsAbs)

BsAbs have been engineered to effectively engage two specific antigens at the same time. These specialized antibodies effectively modulate the immune response by redirecting and stimulating immune cells, blocking the co-inhibitory receptors on these cells, activating molecules that enhance the immune response, interfering with specific signaling pathways, and employing a strategy of simultaneously targeting multiple cancer antigens [365]. BsAbs have been developed for pancreatic cancer treatment, with examples such as anti-EGFR × HER2 [366], anti-CD3 × CEA [367], MCLA-128 (anti-HER2 × HER3 BsAb; zenocutuzumab) [368], anti-CD3 × EGFR BsAb [369], anti-CD3 (Vγ9TCR) × HER2/Neu [370], XmAb22841 (anti-LAG-3 × CTLA-4; NCT03849469), XmAb23104 (anti-PD-1 × inducible co-stimulatory molecule [ICOS]) [371], ATOR-1015 (anti-CTLA-4 × OX40) [372], and KN046 (anti-CTLA-4 × PD-L1) [373]. BsAb targeting CD3 and EGFR-armed activated T cells have the ability to target and kill drug-resistant pancreatic cancer cells. Furthermore, the "priming" of these resistant cells with BsAb-armed activated T cells enhances their responsiveness to chemotherapeutic drugs through modulation of ABC transporter expression [369]. These findings provide insight into the use of BsAbs for immunotherapy against PDAC. A trial tests XmAb23104’s efficacy and safety in treating advanced solid tumors, both alone and with ipilimumab (NCT03752398). The study showed that XmAb23104 was generally well tolerated at doses up to 15 mg/kg in subjects with advanced solid tumors. Clinical activity was observed, including partial responses in three subjects and stable disease for over 12 months in two subjects [371].

### Cancer vaccines

There are several types of cancer vaccines, including whole tumor cell vaccines, DC vaccines, peptide vaccines, DNA vaccines, and mRNA vaccines. While conventional immunotherapies may demonstrate efficacy against cancers featuring identifiable surface antigens specific to tumors, cancer vaccines possess the capability to also encompass a wider range of intracellular antigens for targeting purposes. Up to this point, the FDA has granted approval to a solitary therapeutic vaccine for cancer treatment, namely sipuleucel-T (PROVENGE). This particular vaccine solely enhances patient survival in prostate cancer cases by a mere 4 months [374].

Vaccination is being examined to activate or enhance pre-existing immune responses using agents like GVAX (pancreatic cell lines modified with GM-CSF) or CRS207 (live attenuated *Listeria monocytogenes* expressing MSLN), either alone or in combination with a mAb targeting the CD40 molecule to activate APCs [41]. In this section, we provide an overview of various types of cancer vaccines and underscore the significant studies conducted with these vaccines (Tables 6, 7).

**Table 6:** Categorization of cancer vaccines in the context of pancreatic cancer therapy

Type	Mechanism of action	Cancer vaccine	References
Whole tumor cell vaccine	Irradiated tumor cells elicit an immune response targeting TSAs or TAAs, which are proteins expressed by tumor cells	Algenpantucel-L (NLG0205)	[375]
		Photothermal nanoparticle-loaded tumor cells	[607]
		GVAX	[41, 381, 608]
DC vaccine	DCs are pulsed with TAAs or TSAs, presenting them to effector T cells, resulting in specific immune responses against tumor cells expressing corresponding tumor antigens	MUC1-pulsed DCs	[383]
		WT1-pulsed DCs	[388]
		α-Galactosylceramide-pulsed DCs	[392]
		MAGE-A3-pulsed DCs	[609]
		Mesothelioma lysate-pulsed DCs	[390]
		DC vaccine plus LAK cells	[391]
		DCs loaded with mRNA encoding CEA	[610]
Peptide vaccine	Epitope, peptide, or protein expressed by pancreatic tumor cells elicits robust immune responses	RAS oncogene-based vaccine: GI-4000, TG01	[611–614]
		HSP-peptide complex-based vaccines: HSPPC-96	[615]
		Survivin-based vaccine: AYACNTSTL	[616]
		VEGFR-based vaccine: VXM01	[404, 617, 618]
		Gastrin-based vaccine: G17DT	[619, 620]
		Telomerase-based vaccine: GV1001	[393, 394]
DNA vaccine	Transferring a DNA into an organism's system with the aim of producing an antigen, which in turn triggers a safeguarding immune response	ENO1 DNA vaccine	[397, 398]
		MUC1-VNTRn	[399]
		Chimeric DNA encoding FAPα and survivin	[400]
mRNA vaccine	The mRNA encoding TSAs, TAAs, and tumor neo-antigens induce a robust immune response	RO7198457 (Autogene Cevumeran)	[401]/NCT04161755
Viral/bacterial vector-based vaccine	This type of vaccine use an immunogenic viral or bacterial vectors to deliver the mRNA encoding TAAs or TSAs	Heat-killed whole cell vaccine of *Mycobacterium Obuense*: IMM101	[621, 622]
		Live-attenuated *Listeria Monocytogenes* encoding MSLN: CRS-207	[41]
		Intracellular delivering Salmonella	[403]
Stem cell-based vaccine	Induction of anti-tumor immunity by oncofetal antigens	iPSC-based cancer vaccine, comprised of autologous iPSCs and CpG	[406]

CEA: Carcinoembryonic antigen, DC: Dendritic cell, ENO1: α-Enolase, FAPα: Fibroblast activation protein alpha, HSP: Heat-shock protein, iPSC: Induced pluripotent stem cell, LAK: Lymphokine-activated killer, MSLN: Mesothelin, MUC1: Mucin-1, TAA: Tumor-associated antigen, TSA: Tumor-specific antigen, VEGFR: Vascular endothelial growth factor receptor, VNTRn: Variable number tandem repeat, WT1: Wilms’ tumor 1

**Table 7:** Key clinical trials of cancer vaccines in pancreatic cancer therapy

Agent	Target_(s)_	Mechanism of action	Combination therapy	Participants	Phase	Outcomes*	Status	NCT identifier/Reference
mRNA-5671/V941	KRAS-G12D, KRAS-G12V, KRAS-G13D, KRAS-G12C	A tetravalent mRNA-based cancer vaccine for targeting oncogenic KRAS mutations in PDAC	Pembrolizumab	70	I	Determining AEs, dose-limiting toxicities, ORR, and changes in the quantity of mutant KRAS specific T cells	Completed	NCT03948763
RO7198457 (Autogene Cevumeran/BNT122)	TSAs and TAAs	A personalized mRNA cancer vaccine delivering mRNA containing specific pancreatic cancer neo-antigens	Atezolizumab + FOLFIRINOX	29	I	Longer median recurrence-free survival in responders (receiving vaccine) compared with non-responders (not receiving vaccine; *P* = 0.003) at an 18-month median follow-up 6% (1/16) and 100% of patients had grade 3 and 1–2 AEs, respectively (n = 16) Vaccine-expanded T cells were up to 10% of all blood T cells	Active, not recruiting	NCT04161755/[401]
ELI-002/ELI-002 2P	KRAS-G12D, KRAS-G12R	A peptide-based cancer vaccine that consists of amphiphile KRAS peptides combined with CpG adjuvant	N/A	25	I	No dose-limiting toxicities Median relapse-free survival = 16.33 months 84% (21/25) patients showed mKRAS-specific T cell responses	Active, not recruiting	NCT04853017/[396]
KRAS peptide vaccine + Poly-ICLC adjuvant	KRAS	A peptide-based cancer vaccine against mutant KRAS	N/A	25	I	Determining drug-related toxicities, changes of IFN-γ producing mutant-KRAS-specific CD8^+^ and CD4^+^ T cells at 5/13/17 weeks, and changes in T cell quality (e.g., memory, exhaustion, poly-functionality, and activation)	Recruiting	NCT05013216
	KRAS-G12C, KRAS-G12V, KRAS-G12D, KRAS-G12A, KRAS-G13D, KRAS-G12R	A cancer vaccine against oncogenic KRAS mutations	Nivolumab + Ipilimumab	30	I	Determining OS, DFS, PFS, ORR, changes of IFN-γ producing mutant-KRAS-specific CD8^+^ and CD4^+^ T cells, and drug-related toxicities	Recruiting	NCT04117087
Personalized synthetic peptide vaccine	TSAs and TAAs	Inducing TSA/TAA-specific effector T-cell immune responses	Imiquimod (TLR7 agonist) + Pembrolizumab + Sotigalimab (APX005M; a CD40 agonist)	150	I	Determining AEs, OS, PFS, RFS, response rate, and changes in neoantigen-specific T cell response	Recruiting	NCT02600949

^*^In cases where the outcomes of the clinical trial have not yet been released, the goals (primary/secondary outcome measures) of the study are mentioned

AEs: Adverse events, APC: Antigen-presenting cell, DFS: Disease-free survival, N/A: Not applicable, ORR: Objective response rate, OS: Overall survival, PFS: Progression-free survival, RFS: Recurrence-free survival, TAA: Tumor-associated antigen, TLR7: Toll-like receptor 7, TSA: Tumor-specific antigen

#### Whole tumor cell vaccines

Utilizing a tumor cell vaccination is a simple and straightforward approach to tumor immunotherapy. The tumor cell vaccination contains both CD4^+^ helper T-cell and CTL epitopes. Algenpantucel-L (NLG0205) is an example of such a vaccine. Results from a phase II study demonstrated that the combination of Algenpantucel-L with the adjuvants gemcitabine and 5-fluoruracil yielded an 86% survival rate at one year, a 51% survival rate at two years, and a 42% survival rate at three years [375]. Nevertheless, a study demonstrated that Algenpantucel immunotherapy did not yield advantages for patients suffering from advanced PDAC, despite following the standard of care, neo-adjuvant chemotherapy, and chemoradiation [376].

To elicit T-cell immune responses against various tumor antigens, scientists manufactured a pancreatic cancer vaccine called GVAX. This particular vaccine, classified as allogeneic, consists of human GM-CSF-secreting whole tumor cells [377–380]. A study's findings reveal that neo-adjuvant and adjuvant GVAX, with or without nivolumab (an anti-PD-1 monoclonal antibody) and urelumab (an anti-CD137 agonist), are safe. Furthermore, treating with GVAX alongside nivolumab and urelumab leads to a remarkable increase in tumor-infiltrating activated effector T cells. This combination also demonstrates efficacy by substantially enhancing DFS in comparison to GVAX with or without nivolumab [381].

#### DC vaccines

DCs that were isolated from the patient's peripheral blood were loaded with tumor-associated antigens (TAAs) or tumor-derived mRNA. After the administration of these vaccines, the modified DCs proceed to the lymph nodes, where they transmit antigens to T lymphocytes and concurrently induce co-stimulatory signals [382]. In a study, DCs were gathered from 7 patients who had stage III/IV pancreatic cancer through the employment of apheresis. Afterwards, these collected DCs underwent the process of being pulsed with MUC-1 peptide. The injection of MUC-1-pulsed DCs in these patients exhibited both safety and efficacy, successfully triggering an immune response towards the MUC-1 [383].

Many pancreatic cancer cells exhibit overexpression of the Wilms’ tumor 1 (WT1). Several studies assessed the effectiveness of using DCs pulsed with WT1 peptides and chemotherapy in treating advanced pancreatic cancer [384–388]. A retrospective multicenter analysis was conducted on 255 patients with pancreatic cancer. These patients were receiving standard chemotherapy and a DC vaccine. This study showed that in patients with pancreatic cancer who received a DC vaccine, a positive erythema reaction at the site of DC vaccine injection was linked to better survival [389].

The impact of using mesothelioma lysate-loaded DCs in combination with FGK45 (a CD40 agonist) was examined in PDAC mice models. This innovative technique provoked a remarkable alteration in the transcriptome of the tumor, involving the suppressive indicators on CD8^+^ T cells, and resulted in a considerable improvement in survival [390]. The administration of lymphokine-activated killer (LAK) cell therapy significantly extended the survival of patients with advanced pancreatic cancer who underwent DC vaccine-based immunotherapy along with gemcitabine. Nevertheless, the use of immunotherapy on its own enhanced the quantity of cancer antigen–targeting CTLs while decreasing the presence of Tregs [391]. In vivo, the induction of anti-tumor immunity against pancreatic cancer is achieved through DC vaccines that have been pulsed with alpha-galactosylceramide [392].

#### Peptide vaccines

The peptide vaccine candidate GV1001 possesses certain noteworthy cell-penetrating peptide characteristics. GV1001 is generated from a peptide derived from a reverse-transcriptase portion of telomerase, or hTERT. In a phase II study, a significant immune response was observed in a majority of patients with advanced pancreatic cancer who received GV1001, and these immune responders had a notably improved median survival compared to non-responders [393]. However, a subsequent phase III clinical trial combining chemotherapy with the GV1001 vaccine did not yield a significant improvement in OS [394]. Another peptide cancer vaccine, KIF20A-66, was also examined in a phase I/II trial and found to be well-tolerated. The mOS and median progression-free survival (mPFS) were reported as 142 days and 56 days, respectively [395]. Recently, the phase 1 study of ELI-002 2P in patients with KRAS-mutated pancreatic cancer demonstrated promising results. ELI-002 2P is a cancer vaccine that specifically targets lymph nodes and consists of three components. These components include modified G12D and G12R mKRAS long peptides, which have been modified with amphiphiles, and an amphiphile-modified TLR9 agonistic CpG-7909 DNA. The therapy was well-tolerated, induced significant T-cell responses, and resulted in biomarker clearance and improved relapse-free survival. These findings suggest that ELI-002 2P has the potential to be an effective treatment option for patients with immunotherapy-recalcitrant KRAS-mutated tumors [396].

#### DNA vaccines

Several studies have shown that DNA vaccines targeting TAAs can effectively prolong survival in mice with PDAC. Targeting α-Enolase (ENO1) with a DNA vaccine has been particularly effective [397]. In addition, combining this DNA vaccine with chemotherapy using gemcitabine has shown improved efficacy against multiple TAAs, including ENO1, glyceraldeheyde-3-phosphate dehydrogenase (G3P), keratin, type II cytoskeletal 8 (K2C8), and far upstream binding protein 1 (FUBP1) [398]. Another DNA vaccine targeting mucin 1-variable number tandem repeat (MUC1-VNTRn) has demonstrated strong cytotoxic effects in both in vivo and in vitro experiments [399]. Furthermore, a chimeric DNA vaccine that targets human fibroblast activation protein alpha (FAPα) and survivin has been shown to reduce immunosuppressive cells and increase TILs, thereby creating a more favorable TME for immune responses against pancreatic tumors [400].

#### RNA vaccines

Personalized cancer vaccines made of mRNA include mRNA that encodes specific tumor-specific antigens (TSAs) and TAAs. Subsequently, APCs take in the mRNA and exhibit the matching peptide antigens, which prompts immune responses encompassing CTLs and memory T cells. RO7198457, also known as BNT122, represents an mRNA-based cancer vaccine that aims to elicit T-cell-triggered immune reactions against tumor neo-antigens. Various clinical studies are scheduled to be conducted among individuals diagnosed with diverse types of cancer, such as pancreatic cancer (NCT04161755 and NCT05968326), solid tumors (NCT03289962), melanoma (NCT03815058), and colon cancer (NCT04486378). However, many of these trials’ results have not been released yet. Next, the outcomes of the clinical trial NCT04161755 are explained.

An mRNA vaccine called autogene cevumeran was generated using uridine mRNA-lipoplex nanoparticles. After the surgical procedure, a combination therapy that included atezolizumab, the mRNA vaccine (with a maximum of 20 neo-antigens per patient), and chemotherapy was conducted. The results indicated that vaccine-enhanced T cells, which accounted for as much as 10% of the total T cells in the bloodstream, experienced re-expansion through a vaccine booster. These re-expanded cells consisted of durable, polyfunctional CD8^+^ T cells that targeted pancreatic cancer neo-antigens. After a median follow-up period of 18 months, patients who exhibited vaccine-enhanced T cells demonstrated a significantly prolonged median recurrence-free survival when compared to the control group [401].

#### Viral/bacterial vector-based vaccines

These cancer vaccines use modified viruses or bacteria as vectors to deliver genetic code for tumor antigens into human cells. The infected cells then produce tumor antigens, which trigger an immune response in the host. The bacterial vector can be used to treat castration-resistant prostate cancer, and the bacterial-based cancer vaccine has shown promising anti-tumor effects in clinical trials [402]. One of the widely recognized cancer vaccines of this kind is CRS207, which is a live attenuated strain of *Listeria monocytogenes* engineered to express MSLN. The utilization of the CRS207 vaccine in individuals diagnosed with metastatic pancreatic cancer has demonstrated promising outcomes in terms of prolonged survival rates while causing minimal detrimental effects to patients [41]. The utilization of an exogenous immunization antigen, administered via *Salmonella* bacteria acting as a vector, effectively redirects the attention of CD8^+^ T cells towards cancer cells within the cytoplasm of tumor cells. Consequently, this approach leads to the complete eradication of pancreatic tumors, the enhancement of anti-tumor immunity, and a significant extension in survival duration, as demonstrated in PDAC mouse models [403]. Moreover, VEGFR-2, a target for anti-angiogenic intervention, is expressed on tumor vasculature. VXM01, an oral tumor vaccine using attenuated *Salmonella* with a VEGFR-2 expression plasmid, was tested in a phase I trial with advanced pancreatic cancer patients. The study found that VXM01 was well tolerated, with no dose-limiting toxicities and significant increases in VEGFR2-specific T effector responses. Vaccinated patients showed reduced tumor perfusion and elevated serum biomarkers indicative of anti-angiogenic activity, which correlated with preexisting VEGFR2-specific T-cell levels [404].

#### Stem cell-based vaccines

The fact that cancer cells and embryonic tissues have several similar cellular and molecular characteristics suggests that we can potentially utilize iPSCs to stimulate anti-tumor responses within cancer vaccines. Indeed, iPSCs share gene expression profiles with tumor cells. The prevention of tumor growth in murine breast cancer, mesothelioma, and melanoma models is achieved by iPSC vaccines. Acting as an adjuvant, the iPSC vaccine effectively hinders the reoccurrence of melanoma and decreases the spread of tumors [405]. Research demonstrates that a cancer vaccine derived from iPSCs stimulates a defensive immune response in a PDAC mouse model. Furthermore, this immune response is linked to heightened CD8^+^ effector and memory T cell reactions against tumor cells, the generation of antibodies specifically targeting cancer cells, and a reduction in immunosuppressive Tregs composed of CD4^+^ T cells [406].

### Strategies based on targeting myeloid cells and CAFs

In this section, our primary objective is to elucidate the therapeutic potential associated with specifically targeting myeloid cells in the context of pancreatic cancer (Table 8).

**Table 8:** Targeted approach towards myeloid cells and cancer-associated fibroblasts in pancreatic cancer clinical trials

Strategy	Target/agent	Cellular target	Combination therapy	Participants	Phase	Outcomes*	Status	NCT Identifier/reference
Targeting immunosuppressive myeloid cells	CCR2, 5/BMS-813160	M-MDSCs	Nivolumab + Gemcitabine + Nab-paclitaxel	40	I, II	Detrmining OS, ORR, PFS, and AEs	Active, not recruiting	NCT03496662
	CXCR2/SX-682	PMN-MDSCs	Nivolumab	20	I	Determining maximum tolerated dose, changes in TME composition, PFS, and OS	Recruiting	NCT04477343
Reprogramming DCs	FLT3L/CDX-301, CD40 agonists/CDX-1140	DCs	Pembrolizumab + Chemotherapy	132	I	Determining safety, tolerability, ORR, clinical benefit rate, duration of response, PFS, OS, pharmacokinetics, and immunogenicity	Completed	NCT03329950
Targeting CAFs	VDR/Paricalcitol	CAFs	Gemcitabine + Nabpaclitaxel	36	I, II	Determining AEs, OS, PFS, and response rate	Active, not recruiting	NCT03520790
			Nivolumab + Albuminbound paclitaxel + Cisplatin + Gemcitabine	10	II	Grade 3–4 AEs (n = 24): thrombocytopenia (76%), anemia (44%), and colitis (12%) Partial response = 19, stable disease = 2, progressive disease = 2, ORR = 83%, and mPFS/mOS = 8.17/15.3 months	Active, not recruiting	NCT02754726/[623]
Inhibition of TGF-β	ATR2/Losartan	CAFs	FOLFIRINOX + Nivolumab + Radiotherapy + Surgery	168	II	Determining R0 resection rate, PFS, OS, pathologic complete response, and AEs	Active, not recruiting	NCT03563248
Inhibition of IL-1β	IL-1β/Canakinumab (ACZ885)	CAFs	Spartalizumab + Nabpaclitaxel + Gemcitabine	10	I	No dose limiting toxicities Grade 3/4 AEs: neutropenia (60%) and anemia (50%), pneumonitis (10%), and no fatal AEs (n = 10) Partial response = 3, stable disease = 5, and progressive disease = 2 1-year OS = 60% Activation of CD8^+^ T cells and increased serum levels of CXCL9/10 in both responder and non-responder patients	Active, not recruiting	NCT04581343/[423, 624, 625]

^*^In cases where the outcomes of the clinical trial have not yet been released, the goals (primary/secondary outcome measures) of the study are mentioned

AEs: Adverse events, ATR2: Angiotensin II receptor, CAF: Cancer-associated fibroblast, DC: Dendritic cell, FLT3L: Fms-related receptor tyrosine kinase 3 ligand, IL-1β: Interleukin-1 beta, M-MDSC: Monocytic myeloid-derived suppressor cell, N/A: Not applicable, ORR: Objective response rate, OS: Overall survival, PFS: Progression-free survival, PMN-MDSC: Polymorphonuclear myeloid-derived suppressor cell, TGF-β: Transforming growth factor-beta, VDR: Vitamin D receptor

#### Targeting macrophages

Therapies that retrain macrophages to engulf and destroy tumor cells may provide a new approach to treating cancer. Antibodies that stimulate the phagocytic program in macrophages were initially found in pancreatic cancer patients treated with anti-CD40 agonistic antibodies [407]. It was believed that these antibodies only affected macrophages, but further research showed they also improved the function of DCs and T-cell priming [408, 409]. However, a phase II trial found that an anti-CD40 antibody called APX005M (sotigalimab) did not improve clinical outcomes in pancreatic cancer patients, suggesting that its mechanism of action may be different in humans [410]. As mentioned earlier, CD47 is a protein found on cancer cells that prevents them from being engulfed by macrophages. However, blocking CD47 alone does not have a significant effect on some types of solid tumors [411]. Macrophages can be stimulated to have anti-cancer properties, including engulfing cancer cells expressing CD47, by using a specific molecule, CpG oligodeoxynucleotide (an agonist for the TLR9) [412]. The utilization of a specific TLR9 ligand known as K3-SPG for in situ vaccination prompts a durable immune response and enhances the effects of both local and systemic immunotherapy in preclinical models [413], which may be associated with overcoming T-cell exhaustion [414]. Moreover, signal transduction by the CSF-1R in macrophages could be a useful target for improving the immune response in pancreatic tumors and enhancing the effectiveness of immunotherapy. Blocking the CSF1/CSF-1R pathway eliminates TAMs from tumors and reprograms remaining macrophages to enhance anti-tumor immunity. This blockage improves interferon responses, increases infiltration of CTLs, and prevents tumor growth [159]. In a trial investigating the safety and immunologic impact of GVAX in combination with cyclophosphamide, pembrolizumab, and IMC-CS4 (a CSF-1R inhibitor), nine patients were enrolled, with two experiencing severe immune-related side effects (diarrhea and rash). The study reported a median DFS of 12.6 months and OS of 20.4 months, with 78% achieving major pathological response post-surgery. The primary immunologic endpoint was met, with 75% of patients showing a significant increase in CD8^+^ T cells and granzyme B^+^ CD8^+^ T cells following triple therapy. No significant change in myeloid cell density was observed, suggesting macrophages were reprogrammed rather than depleted (NCT03153410) [415].

PDAC is a type of cancer that spreads to the liver with the help of macrophages. The process of macrophages engulfing dead cells, known as efferocytosis, promotes liver metastasis by changing the macrophages. A protein called progranulin in macrophages affects their ability to break down cells, leading to a change in the macrophages and an increase in arginase 1 levels. Blocking efferocytosis or reducing progranulin levels can decrease liver metastasis and enhance the function of CD8^+^ T cells [416]. Targeting these mechanisms may prevent the spread of PDAC to the liver.

#### Targeting CAFs

Although previous attempts to target CAFs in PDAC have failed, there is renewed interest in targeting subgroups of fibroblasts or their secreted products. Schwann cells provoke CAFs in the microenvironment of PDAC [417]. Suppressing stromal TGF-βR2 leads to a decrease in IL-6 production from CAFs, which in turn results in diminished STAT3 activation in tumor cells and a reversal of the immunosuppressive environment [418]. Also, In vivo neutralization of TGF-β remodels CAF dynamics, reducing myofibroblasts and promoting interferon-responsive fibroblasts. This enhances anti-tumor immunity and the effectiveness of PD-1 immunotherapy [419].

It is suggested that vitamin D might play a role in inducing a state of rest or inactivity in fibroblasts. A study indicates that the stroma of human pancreatic tumors contains the vitamin D receptor (VDR). Using calcipotriol, a ligand of VDR, as a treatment significantly reduces inflammation and fibrosis in both pancreatitis and tumor stroma. The study demonstrates that VDR plays a crucial role as a transcriptional regulator of PSCs, aiding them to revert to a dormant state. This results in stromal alterations, enhanced intratumoral delivery of the chemotherapy drug gemcitabine, a decrease in tumor size, and a survival rate increase of 57% compared to chemotherapy alone [420]. In light of this finding, two phase II clinical trials are currently in progress for patients with PDAC. The first trial (NCT03520790) aims to combine paricalcitol with gemcitabine and nab-paclitaxel, while the second trial (NCT02754726) seeks to combine nivolumab with gemcitabine, paclitaxel, and cisplatin. These trials are actively recruiting participants to further investigate the potential benefits of these treatment approaches.

IL-1β has multiple effects on the TME, including promoting the development of CAFs. These CAFs, in turn, produce IL-6, which creates an environment that helps tumors evade the immune system and allows tumor cells to survive for longer [231, 421]. Not only immune cells, but PDAC tumor cells themselves can also produce IL-1β. In preclinical studies, blocking IL-1β has shown promising results when combined with blocking PD-1 [422]. A phase I clinical trial (NCT04581343) evaluated the efficacy of combining gemcitabine and nab-paclitaxel with two antibodies—one that blocks IL-1β (canakinumab) and another that blocks PD-1 (spartalizumab). The most common severe AEs (Grade 3/4) included neutropenia (60%) and anemia (50%), with no fatalities. One patient discontinued spartalizumab due to grade 3 pneumonitis. There were 3 confirmed partial responses, 5 patients with stable disease, and 2 patients with disease progression as their best response (n = 10), and a 1-year OS rate of 60% was reported. Both patients who responded and those who did not showed CD8^+^ T cell activation in peripheral blood and increased serum levels of IFN-induced chemokines CXCL9/10 [423].

CAFs have elevated levels of the protein PIN1. PIN1 promotes several cancer-related pathways by affecting the structure of phosphorylated proteins. Blocking PIN1 with drugs has shown promise in treating cancer. Several small molecule drugs, such as all-trans retinoic acid (ATRA), have been identified as PIN1 inhibitors and have been used to study their functions in cancer development [424, 425]. Clinical trials have shown positive results when combining ATRA with chemotherapy in patients with advanced pancreatic cancer [426]. PIN1 inhibition also reduces the formation of fibrous tissue within tumors and increases sensitivity to chemotherapy drugs. In addition, PIN1 inhibition may enhance the effectiveness of immunotherapy [425, 427]. Animal models have demonstrated reduced tumor growth when treated with a combination of a PIN1 inhibitor AG17724, an antibody against FAPα, and DNA aptamers that recruit specific immune cells [424].

Placental growth factor (PlGF) is a protein expressed mainly in the placenta. Blocking PlGF in animal models of intrahepatic cholangiocarcinoma led to improved survival by decreasing desmoplasia and enriching quiescent CAFs [428]. PlGF also promotes liver fibrosis, tumor angiogenesis, and cancer cell metastasis [429]. In pancreatic cancer, PlGF is upregulated by chemotherapy, leading to the generation of extracellular matrix by CAFs. Combining atezolizumab (an anti-PD-L1 mAb) with PlGF/VEGF inhibition targeting CD141^+^ CAFs enhances the efficacy of chemotherapy [429].

#### Reprogramming DCs

The restoration of the expression of peptide-MHC complexes and co-stimulatory molecules is achieved through DC reprogramming. This reprogramming enabled the display of tumor antigens originating within the context of MHC-I, ultimately enhancing the targeted elimination by CD8^+^ CTLs [430]. Studies in mice have demonstrated that cDCs play a crucial role in initiating immune responses specific to tumors by CD8^+^ T lymphocytes. However, pancreatic cancer lacks an adequate presence of these cDCs [130, 431]. In comparison to lung adenocarcinoma mouse models, models of PDAC displayed a notable scarcity of CD103^+^ cDCs. Following treatment with FMS-like tyrosine kinase 3 ligand (FLT3L), which augments the number of intratumoral cDCs, mouse models of PDAC showed renewed sensitivity to CD40 agonist antibody and radiation therapy [431]. Currently, a trial is being conducted to investigate the potential of combining CDX-1140, a CD40 agonist antibody, with FLT3L (CDX-301) in patients with PDAC (NCT03329950).

The activation of DCs can be provoked by the death of tumor cells, and the subsequent ingestion of fragments from these tumor cells also triggers regulatory processes in DCs that hinder their interaction with T cells. By subjecting DCs to microbial products that stimulate TLR signaling, such as pIpC or CpG DNA, which imitate viral nucleic acid, this regulatory function can be bypassed. Hence, innate immune adjuvants are incorporated into vaccination strategies. Additionally, targeted medications that affect the pathways of DNA replication and repair can activate the STING pathway, which subsequently stimulates the production of IFN-I and enhances the activation of DCs [432, 433]. As a result, in animal models of pancreatic cancer, the administration of STING agonists boosts inflammation in the surrounding immune environment and reduces tumor load [434]. Moreover, using Pt^IV^-MSA-2 conjugates containing cisplatin and a STING agonist is effective against pancreatic cancer, leading to increased immune cell infiltration and activation in tumor tissues [435].

The enforcement of expression of the transcription factors PU.1, IRF8, and BATF3 (PIB) is adequate to trigger the cDC1 phenotype. By this reprogramming through PIB, cancer cells are transformed into capable APCs, offering an approach to counteract the strategies employed by tumors to evade immune surveillance. These reprogrammed DCs were capable of presenting endogenous tumor antigens on MHC-I and facilitating targeted killing by CD8^+^ T cells [430].

#### Targeting immunosuppressive MDSCs

There is significant synergy between PD-1 blockade and the CD11b agonist as it substantially decreases the accumulation of the majority of myeloid cell types in PDAC mice models [436]. Furthermore, CD11b agonists like GB1275 cause reprogramming of the innate immune system, leading to an enhanced response of pancreatic cancer to immunotherapies [341]. Mice that were administered CCR2 inhibitors specifically aimed at circulating monocytes experienced a reduction in the PDAC tumor load [153]. A study found the ideal dose of the CCR2 inhibitor PF-04136309 to be used alongside chemotherapy in a clinical trial for pancreatic cancer patients. The combination therapy was found to be safe and well-tolerated by the patients. The study also discovered that the inhibitor caused monocytes to accumulate in the bone marrow of patients, leading to a decrease in circulating monocytes and M-MDSCs in the TME. This resulted in significant reductions in the size of the primary tumors [437]. Nevertheless, the combination of nab-paclitaxel/gemcitabine along with PF-04136309 resulted in notable pulmonary toxicity and failed to demonstrate a favorable indication [438]. The signaling of CXCR2 is found to be increased in myeloid cells. The absence of CXCR2 leads to a decrease in metastasis and its inhibition extends the period of survival without tumors in mice. Also, the suppression of CXCR2 improves the infiltration of T cells and makes them more responsive to anti-PD-1 therapy [439].

## Gut microbiome in modulating immune checkpoint blockade

Preclinical research on mice with sarcoma, melanoma, and colon cancer revealed that the most effective responses to anti-CTLA-4 and anti-PD-L1 treatments were reliant on the existence of certain species of gut bacteria. This underscores the connection between the gut microbiome and the success of immune checkpoint therapy [440–444]. The connection between certain gut bacteria and the immune response has also been noted in individuals with cancer. These bacteria impact the operation and maturation of immune cells in lymph nodes or within the TME, thus dictating the success of ICB [445]. Specific gut bacteria have been identified to affect immune responses in cancer. For instance, Bacteroides fragilis can trigger TH1 responses and assist in the development of DCs in tumors, enhancing the effectiveness of anti-CTLA-4 treatment. Bifidobacterium can modify the activation of DCs and amplify the activity of CD8^+^ T cells that are specific to the tumor. Akkermansia muciniphila can augment the penetration of particular CD4^+^ T cells into tumors and elevate the proportion of effector to regulatory CCR9^+^CXCR3^+^CD4^+^ T cells [22, 440, 441, 446].

The fact that pancreatic cancer tissues contain a unique microbial fingerprint that aids in acquiring cancer characteristics and influences the long-term survival of patients has now been widely accepted and acknowledged [447–449]. Pancreatic cancer progression and the effect of particular treatments have been linked to separated alterations observable in the microbiome of the gut and tumor [442, 450, 451]. For instance, a tryptophan metabolite derived from tryptophan by gut microbiota, known as indole-3-acetic acid (3-IAA), is linked to improved responses to treatment. Alterations in diet or the administration of 3-IAA enhanced the effectiveness of chemotherapy in mouse models of PDAC. This effectiveness is associated with myeloperoxidase, which oxidizes 3-IAA, resulting in an increase in ROS and a decrease in autophagy in cancer cells, thereby inhibiting their proliferation [452]. In another study, the oncogenic mutation KRAS G12D boosts IL-33 production, promoting type 2 immunity in PDAC. The tumor’s mycobiome further increases IL-33 secretion. This IL-33 then recruits and activates TH2 cells and innate lymphoid cells 2 (ILC2s) in the PDAC TME. Remarkably, either deleting IL-33 genetically or administering anti-fungal treatment leads to PDAC tumor regression [453]. This highlights the crucial roles of IL-33 and the tumor’s mycobiome in PDAC progression and potential treatment strategies.

Multiple studies conducted on murine models of PDAC have demonstrated that eliminating the gut microbiome with antimicrobial agents could potentially amplify the susceptibility of tumors to ICIs and diminish the overall burden of tumors [70, 454, 455]. While the majority of the literature concentrates on the potential employment of microbiota-centered interventions together with chemotherapy and ICB, it emerges as plausible that microbiome modulation may also be employed concurrently with CAR T cells, antibody–drug conjugates, and immunotherapies that are yet to be established [456]. A multitude of current research and clinical trials are exploring the possibility of modifying the microbiome to boost the efficacy of ICB. It has been demonstrated that fecal microbial transplantation can enhance ICB outcomes and reduce associated AEs in patients [457–459]. Furthermore, altering the diet is a potential approach to adjust the gut microbiome, and an increasing number of preclinical studies indicate its potential to enhance the response to ICB [460].

A study suggests that the composition of the tumor microbiome in resected pancreatic adenocarcinoma patients plays a significant role in long-term survival. The higher alpha-diversity in the tumor microbiome of long-term survivors and the identified microbiome signature predictive of long-term survivorship highlight the importance of the tumor microbiota in influencing the natural history of the disease. Furthermore, the findings from fecal microbiota transplantation experiments demonstrate the ability to modulate the tumor microbiome and affect tumor growth and immune infiltration, indicating the potential for targeted interventions to improve patient outcomes [459]. Moreover, in individuals with pancreatic cancers, a higher presence of Megasphaera within the tumor has been linked to improved survival rates following anti-PD-1 therapy [461]. Additionally, bacterial elimination in PDAC leads to immune changes, reducing MDSCs, promoting CD8^+^ T-cell activation, and increasing differentiation of TH1 cells and M1 macrophages. This also enhances immunotherapy effectiveness by increasing PD-1 expression. The PDAC microbiome induces TLRs driven-T cell anergy, suggesting the microbiome’s role in immune suppression and its potential as a therapeutic target [70].

All in all, the association of pancreatic cancer with gut and tumor microbiome in the context of cancer immunotherapy is an interesting research area in treatment, prognosis, and predicting response to immunotherapy. Approaches like modulating the gut microbiome, fecal microbial transplantation, and dietary regimen-oriented interventions might improve the clinical outcome in patients undergoing cancer immunotherapy.

## CRISPR/Cas9 and pancreatic cancer immunotherapy

**Fig. 5:**
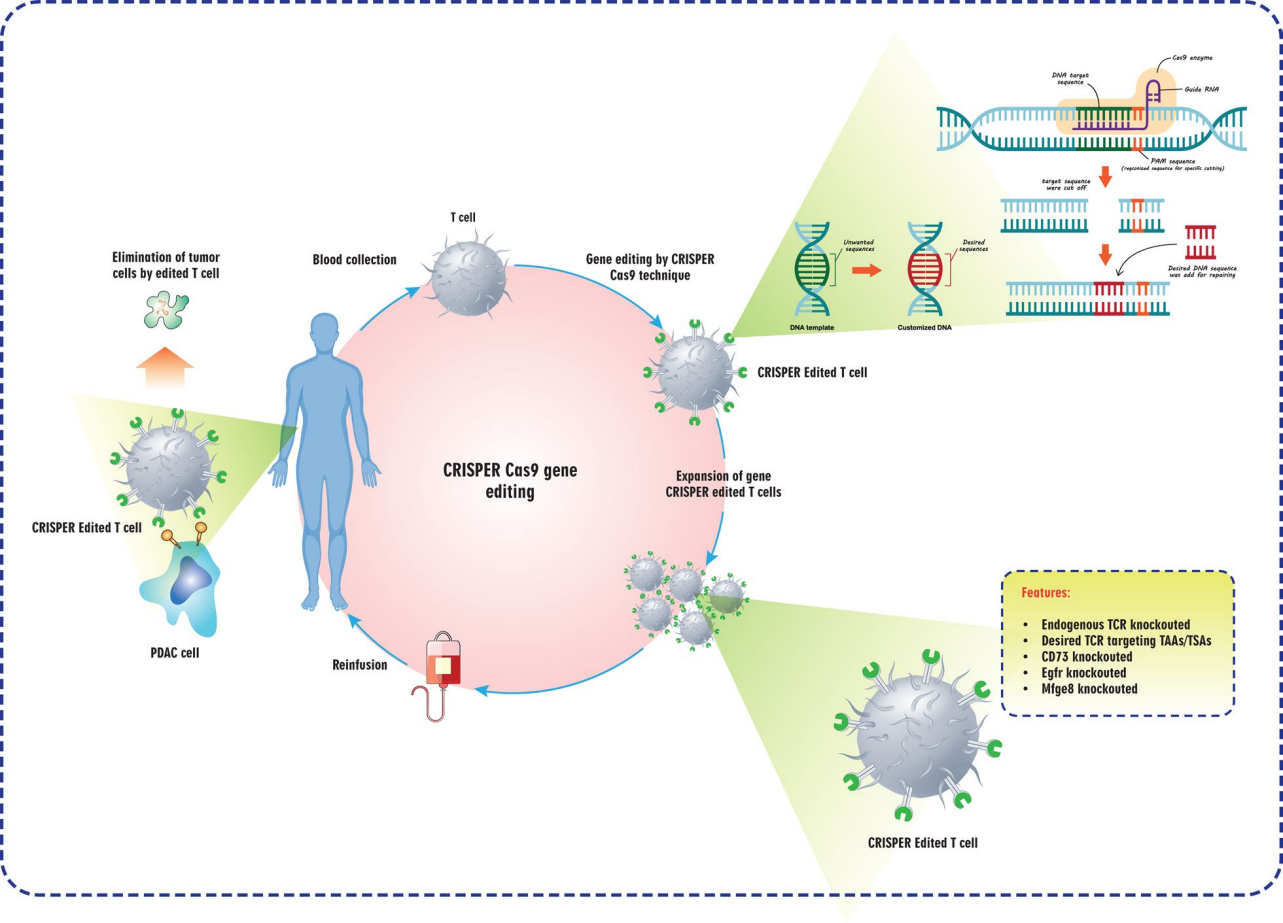
The emerging role of genome editing technology CRISPR/Cas9 in pancreatic cancer treatment. Utilizing CRISPR/Cas9 technology, autologous T cells are genetically modified to eliminate or alter genes that contribute to T cell exhaustion or resistance to immunotherapy. Once modified, these cells are reinfused into the patient, effectively improving the eradication of pancreatic ductal adenocarcinoma (PDAC) cells. TAA: Tumor-associated antigen; TCR: T cell receptor; TSA: Tumor-specific antigen

CRISPR/Cas9, a precise gene editing tool, is revolutionizing cancer research and treatment. The combination of CRISPR/Cas9 and cancer immunotherapy may further broaden the application of immunotherapy to more cancer patients, and ongoing clinical trials are using the CRISPR/Cas9 system in immune cells to modify genomes in a target-specific manner. The CRISPR/Cas9 system's ability to create site-specific, highly efficient gene knockout makes it a desirable tool to address long-standing challenges in cancer treatment, such as T cell exhaustion and TME immunosuppression [462, 463]. In the context of pancreatic cancer, there are numerous studies utilizing CRISPR/Cas9 for gene knockout (Fig. 5) [464–468]. The utilization of CRISPR/Cas9 methodology to disrupt the CD73 gene in both human and murine cellular models of pancreatic cancer demonstrated that CD73 inactivation impeded cellular proliferation and motility, leading to a halt in the G1 phase of the cell cycle. Additionally, it was observed that deletion of CD73 hindered the ERK/STAT3 signaling pathway while stimulating the E-cadherin pathway [469]. A study found that mesenchymal-like pancreatic cancer cells are more resistant to immune cell-mediated killing than the parental epithelial-like cells. In this study, the researchers used CRISPR-Cas9 knockout screens to identify the genes involved in this resistance. They discovered several mesenchymal-specific regulators, such as Egfr and Mfge8, that were responsible for inhibiting immune cell function [470]. The application of CRISPR/Cas9 technology to introduce targeted BRCA1/2 mutations enables the reinstatement of olaparib responsiveness in pancreatic cancer cells [471]. Apart from potential challenges and limitations of CRISPR/Cas9 such as off-target toxicity, Cas9-related immunogenicity, and off-target mutations, these studies highlight the useful application of this genome editing tool in the context of pancreatic cancer immunotherapy.

## Potential strategies improving efficacy of pancreatic cancer immunotherapies

### Combination therapy

Pancreatic cancer presents a range of mechanisms that resist immunotherapy. Therapies that target only one mechanism have not yielded successful results. The research proposes the optimization of the benefits of current agents through logical combinations. The suggested approach for advanced immunotherapy for PDAC involves combinations that amplify immune activation, inhibit immune checkpoints, improve the TME, and are compatible with conventional cytotoxic therapy [472, 473]. There are a plethora of combination therapies for cancer immunotherapy of PDAC (Tables 5, 7, 8, 9). For example, BMS-687681, which acts as a dual antagonist for CCR2/5, was used in conjunction with anti-PD-1 and radiotherapy. The results indicated an increase in the infiltration of intratumoral effector and memory T cells, while simultaneously observing a decrease in the infiltration of Tregs, M2 TAMs, and MDSCs [474]. Overall, combination therapies could improve the efficacy of immunotherapies due to providing a potential for synergistic effects.

**Table 9:** Key combination therapies of PDAC

Immunotherapeutic strategy	Immunotherapeutic agents	Combination therapy	Study design	Results	References
Cancer vaccine and ICB	GVAX cancer vaccine and nivolumab	Urelumab (anti-CD137 [4-1BB] agonist)	Murine syngeneic model of metastatic PDAC	Activated, effector memory T cells ↑ Conversion of T cells from an exhausted status to an activated status ↑ Expression of costimulatory molecules CD137 and OX40 on T cells ↑ Expression of IFN-γ in EOMES^+^ exhausted TILs Survival ↑	[626]
ICB	Anti-PD-1 antibody	Az (a histamine receptor H1 antagonist)	Pancreatic cancer cell specimens from PDAC patients Orthotopic models	Upregulation of MHC-I expression in tumor cells via cholesterol biosynthesis signaling ↑ CD8^+^ CTLs cell penetration and efficacy ↑ Resistance to ICB ↓	[627]
ICB	Anti-PD-1 antibody	Anti-IL-8 antibody	Humanized mouse model of PDAC	Anti-tumor activity of anti-PD-1 ↑ Number of infiltrating granulocytic myeloid cells in TME ↑ Type I interferon production by infiltrating granulocytic myeloid cells ↑	[628]
ICB	Anti-PD-1 antibody	Focal adhesion kinase inhibitor (FAKi), PEGylated recombinant human hyaluronidase (PEGPH20), and anti-CXCR4 antibody	Murine liver metastasis syngeneic model of PDAC	Survival ↑ T-cell infiltration ↑ Number of effector memory T cells ↑ Number of MDSCs ↓ Number of CXCR4-expressing myeloid cells (granulocytes) ↓ Liver metastasis ↓	[484]
ICB	Nivolumab	GVAX cancer vaccine, radiotherapy, and BMS-687681 (dual antagonist of CCR2 and CCR5)	PDAC mouse models	GVAX and nivolumab-induced CCR2/CCR5 expression Intratumoral effector and memory T cell infiltration ↑ Expression of CCL17/CCL22 chemokines ↑ Infiltration of Tregs, M2-like TAMs, and monocytic-MDSCs ↓ Expression of immunosuppressive CCL2/CCL5 chemokines ↓ Anti-tumor efficacy and survival ↑ No improvement of anti-tumor activity by adding GVAX	[474]
ICB	Anti-PD-1 antibody (clone RMP1-14)	Gemcitabine-conjugated polymer (PGEM; a STING agonist) loaded with PF-6309 (a CCR2 antagonist)	Pancreatic tumor spheroid model and orthotopic tumor model	Pancreatic tumor burden ↓ Sensitization of PDAC tumors to anti-PD-1 therapy ↑ Providing anti-tumor immunity through reversing the CCL2/CCL7-mediated immunosuppression	[629]
ICB	Anti-PD-1 and anti-CTLA-4	IACS-8803 (a STING agonist)	Orthotopic Kras^+/G12D^ TP53^+/R172H^ Pdx1-Cre (KPC) derived models PDAC	Inflammatory remodeling of the PDAC stroma ↑ Survival ↑ Sensitivity to ICB ↑	[495]

CTL: Cytotoxic T lymphocyte, CTLA-4: Cytotoxic T-lymphocyte associated protein 4, ICB: Immune checkpoint blockade, MDSC: Myeloid-derived suppressor cell, PD-1: Programmed cell death protein 1, PDAC: Pancreatic ductal adenocarcinoma, STING: Stimulator of interferon genes, TAM: Tumor-associated macrophage, TIL: Tumor-infiltrating lymphocyte, Treg: Regulatory T cell, ↑: Increase, ↓: Decrease

### Costimulatory molecule agonists

CD40 activation and using CD40 agonists are a novel clinical opportunity for cancer immunotherapy [407, 475–478]. There are several agonistic anti-CD40 antibodies, such as SGN-40, SEA-CD40, selicrelumab, APX005M, CDX-1140, and ADC1013, applicable in clinical trials [477]. In a phase I clinical trial, the combination of an agonistic anti-CD40 antibody and gemcitabine for treating PDAC was tested. The treatment showed only a slight effect, but its safety was confirmed [479]. The combination of the CD40 agonist and gemcitabine could potentially overcome resistance to anti-PD-1/CTLA-4 therapy by increasing the accumulation of CD8^+^ T cells that fight against tumors in PDAC [480]. A study found that selicrelumab (an agonist CD40 antibody) significantly altered the TME in PDAC patients. Selicrelumab-treated tumors were enriched with T cells (82%) compared to untreated (37%) and chemotherapy/chemoradiation-treated tumors (23%). Additionally, selicrelumab reduced tumor fibrosis, decreased M2-like tumor-associated macrophages, and matured intratumoral DCs. The treatment had an acceptable toxicity profile and resulted in an overall survival of 23.4 months [475]. Moreover, a study demonstrates that using a nanofluidic drug-eluting seed (NDES) for sustained, low-dose intratumoral delivery of CD40 monoclonal antibody can alter the TME and reduce tumor size in mouse models of PDAC [478]. These findings elucidate the therapeutic mechanisms of CD40 targeting and modification of the TME in pancreatic cancer, aiming to enhance the effectiveness of immunotherapies against cold tumors like PDAC.

### Neutralizing tumor acidity

Acidosis plays a significant role as an immunosuppressive mechanism that contributes to the proliferation of PDAC and immune escape [481]. A study investigates the application of L-DOS47, a urease immuno-conjugate, for the purpose of neutralizing the acidity of tumors and enhancing the response to immunotherapy. L-DOS47 attaches to CEACAM6, a protein that is predominantly present in gastrointestinal cancers, and increases the local pH by breaking down urea into two NH4 + and one CO2. This was experimented on a model of pancreatic tumors in mice, and it was observed that L-DOS47 elevated the extracellular pH of the tumor. When L-DOS47 was used in conjunction with anti-PD-1, it significantly boosted the effectiveness of the monotherapy, leading to a reduction in tumor growth for a duration of up to 4 weeks [482]. This study paves the way for using L-DOS47 in future clinical trials.

### Targeting desmoplastic barriers of TME

A significant obstacle to the effectiveness of cancer immunotherapies in PDAC is the presence of desmoplastic barriers within the stromal ECM, such as hyaluronan. These mechanical barriers encapsulate the tumor cells, thereby restricting their exposure to immunotherapeutic agents. The targeted removal of hyaluronan in a mouse model of PDAC resulted in better vascular permeability and enhanced drug delivery. This led to increased effectiveness of chemotherapy when combined with the cytotoxic chemotherapy drug, gemcitabine [483]. A study found that combining PEGPH20 (a PEGylated recombinant human hyaluronidase), focal adhesion kinase inhibitor, and anti-PD-1 antibody treatments improved survival in PDAC-bearing mice, increased T-cell infiltration, altered T-cell phenotype and metabolism, reduced granulocytes, and decreased CXCR4-expressing myeloid cells. Additionally, adding an anti-CXCR4 antibody significantly reduced metastatic rates in a PDAC liver metastasis model [484].

### Innate immune activation

A useful strategy in combating pancreatic cancer involves the stimulation of the body’s innate immune system to bolster its anti-cancer defenses. This is accomplished by, for instance, utilizing a genetically altered version of *Listeria monocytogenes*, which is engineered to produce MSLN. Alongside this, a vaccine named GVAX is used. The synergistic effect of this combination has been shown to enhance the survival rates of patients [41, 378, 485]. This therapeutic approach transforms PDACs into a state that is more receptive to immune responses. This change is marked by a rise in T cell infiltration and the formation of tertiary lymphoid clusters within the tumors [380], likely converting the cold tumor into the hot tumor. Innate immune cells use cGAS to trigger inflammatory signals when they bind to the pathogen or damage-related molecular patterns (PAMPs/DAMPs). This process leads to the production of cGAMP and the activation of STING, a protein in the endoplasmic reticulum, which then promotes cellular gene programs resulting in the production of IFN-I [486, 487]. IFNs-I (IFN-α and IFN-β) are essential for the development of CD8^+^ T cells that fight against tumors. Tumors that are inflamed with T cells, often referred to as “hot” tumors, have been linked to a transcriptional signature of type 1 interferon [488, 489]. The activation of STING, either systemically or within the tumor, through STING agonists, has been shown to reverse immune-suppression and cause tumor shrinkage in various preclinical cancer studies [434, 490–494]. IACS-8803 (a STING agonist) increases sensitivity to anti-PD-1 and anti-CTLA-4 immunotherapy in the orthotopic PDAC models [495]. Furthermore, a STING agonist called IMSA101 boosts CAR T cell function in a mouse pancreatic tumor model, which is facilitated through STING agonist-induced IL-18 secretion [496]. All in all, the STING innate immune sensing pathway, when activated, could potentially transform tumors lacking T cell infiltrates into tumors with infiltrating T cells, and thus offers a promising target in PDAC immunotherapy.

### TME-modulating agents

A successful immunotherapy for PDAC usually requires combining different treatments to help T cells infiltrate and stay activated in the hostile TME. Current research is focused on developing strategies to improve the PDAC TME, boost the immune response, and enhance the effectiveness of T cell therapy [44, 497].

ADH-503 is a small molecule that binds to CD11b and enhances the adhesion of myeloid cells, inhibiting their migration into tissues [498]. It also shifts TAM polarization to an anti-tumor phenotype, improves survival in PDAC-bearing mice, and sensitizes PDAC tumors to anti-PD-1/PD-L1 immunotherapy [341]. In clinical trials, ADH-503 was well tolerated with common side effects, but no clinical responses were observed in pancreatic cancer patients (NCT04060342) [499].

In PDAC, it is expected that targeting the CCL2/CCR2 pathway would help to reduce the accumulation of TAMs in the TME. A CCR2 inhibitor, PF-04136309, was tested in combination with chemotherapy in patients with pancreatic cancer. A manageable level of safety was observed. Early correlational studies indicated a decrease in TAMs and an increase in TILs (NCT01413022) [437]. Another trial combining PF-04136309 with chemotherapy in pancreatic cancer patients showed high rates of lung toxicity and no significant improvement in efficacy (NCT02732938) [438]. A study tested the CCR2 antagonist CCX872-B in combination with FOLFIRINOX for patients with advanced pancreatic cancer. Analysis showed an OS rate of 29% at 18 months with no safety concerns (NCT02345408) [500]. BMS-813160 is a dual antagonist for CCR2 and CCR5 that is being tested in combination with chemotherapy or immunotherapy in patients with advanced pancreatic or colorectal cancer, but no results from the study have been reported yet (NCT03184870) [501].

Several studies have shown that by inhibiting the CXCR4/CXCL12 axis, the PDAC TME can be modified. For example, when CXCR4 was knocked down, the invasion potential of pancreatic cancer cells in vitro was decreased. Treating fresh human PDAC slices with a combination of PD-1 and CXCR4 blockade resulted in enhanced tumor cell death and lymphocyte expansion into the juxtatumoral compartment [502]. In a mouse model of PDAC, administering the CXCR4 inhibitor AMD3100 (plerixafor) led to the accumulation of T cells among cancer cells, resulting in a synergistic tumoricidal effect when combined with anti-PD-L1 immunotherapy [35]. A trial tested AMD3100 in patients with colorectal and pancreatic cancer, resulting in decreased tumor markers (circulating tumor DNA and IL-8) and changes in immune cells (reduced number of CAFs and increased number of effector TILs/NK cells) [503–505]. Motixafortide (BL-8040; CXCR4 antagonist) is a synthetic peptide that is administered subcutaneously and has shown promising results in combination with pembrolizumab in treating metastatic PDAC, increasing CD8^+^ T cell infiltration and decreasing MDSCs and circulating Tregs (NCT02826486) [506]. NOX-A12 (olaptesed pegol) is a PEGylated drug that inhibits CXCL12 and enhances the activity of anti-PD-1 therapy in pre-clinical models [507]. In a phase 1/2 study with advanced PDAC patients, NOX-A12 in combination with pembrolizumab led to induced TH1 cytokines, prolonged stable disease, and increased effector immune cells in tumor biopsy tissue (NCT03168139) [508].

Pharmacologic inhibition of the A2A adenosine receptor enhances the effectiveness of anti-PD-1 therapy [509]. Several anti-CD73 therapeutics and adenosine receptor inhibitors have been developed [510]. Oleclumab (MEDI9447), a monoclonal antibody that targets CD73, demonstrated positive outcomes in inhibiting tumor progression and promoting immune cell infiltration in colon cancer models. When used in conjunction with anti-PD-1 treatment, it resulted in the elimination of tumors in 60% of animal subjects [511]. Clinical studies of oleclumab, either alone or in combination with durvalumab, in patients who did not respond to anti-PD-L1 therapies like advanced pancreatic cancer revealed good tolerability and some partial responses (22 and 28 months) in a small subset of patients (2/73; NCT02503774) [512]. A study evaluated the safety of quemliclustat (a small molecule inhibitor of CD73) in combination with standard treatment and zimberelimab in patients with metastatic PDAC. The safety profile is similar to single agents, with no new toxicities. Some patients showed partial responses with long-lasting effects (NCT04104672) [513].

## Main challenges ahead of pancreatic cancer immunotherapy

Immunotherapy for pancreatic cancer is a significant therapeutic strategy. However, despite comprehensive studies, there are obstacles in translating research outcomes and determining the best therapeutic combinations. These challenges necessitate a joint effort from scientists and medical practitioners to deepen our comprehension of the interactions between cancer and the immune system and to enhance the treatment choices available to patients. In this part, we will explore in greater detail the chief hurdles facing immunotherapeutic strategies for pancreatic cancer.

### Low antigenic strength and number of neo-antigens

During the process of tumor development, non-synonymous gene mutations occur, leading to the generation of neo-antigens that are exclusively expressed by tumor cells. Pancreatic cancers carry a moderate load of these non-synonymous neo-antigenic mutations [497]. In essence, PDACs show a low load of neo-epitopes; therefore, the tumors are more likely to adapt to immune pressure and escape T cell-mediated killing through cancer immunoediting. In a study, T cell immunity was assessed in a mouse model of pancreatic cancer, revealing a low level of mutations, no anticipated neo-epitopes resulting from these mutations, and resistance to respond to checkpoint immunotherapy [514]. Also, pancreatic tumors that have the greatest quantity of neo-antigens and the highest concentration of CD8^+^ T cell infiltrates are linked to the longest survival rates in patients. Moreover, enrichment of neo-antigens in the tumor antigen MUC16 (CA125) was observed in long-term survivors of pancreatic cancer [53]. Thus, the quality of neo-antigens as a biomarker for PDAC could potentially steer the use of immunotherapies [53, 515]. Additionally, the BCL2A1 neo-epitope is presented as a potential target for personalized immunotherapy, which stimulates CTLs to combat pancreatic cancer cells [516]. All in all, neo-antigens might broaden the horizon towards personalized immunotherapy of pancreatic cancer. A major challenge restricting their applicability, however, is that a low number of neo-antigens is rarely shared among patients [497], making the use of relevant treatment approaches cumbersome and costly.

### Primary, adaptive, and acquired resistance

In primary resistance, there can be instances where cancer does not respond to immunotherapy, potentially due to adaptive immune resistance mechanisms. Adaptive resistance pertains to a resistance strategy where the cancer, even though identified by the immune system, shields itself by adjusting to the immune attack. Lastly, acquired resistance refers to a situation where a cancer initially shows a response to immunotherapy, but after a certain period, it experiences a relapse and advances [517]. From a biological perspective, resistance to immunotherapy can be linked to both intrinsic factors in tumor cells and extrinsic factors associated with TME like ECM and stroma-derived factors, immune cells/factors, and intratumoral microbiota. Tumor cell-intrinsic factors include genetic/epigenetic defects, IFN-γ signaling, lack of neo-antigens, oncogenic signaling pathways, and epigenetic reprogramming [22]. FAP^+^ CAFs hinder the anti-tumor activity of T cells in pancreatic cancer. However, directing therapies towards these FAP^+^ subtypes improves the tumor’s response to anti-PD-L1 [35]. T-cell exclusion is a process that resists immune checkpoint therapy, and it’s particularly noticeable in ‘cold’ tumors like pancreatic cancer, which have a low presence of T cells in TME. Some cancer-causing pathways might allow tumors to use this method to avoid the immune system. For instance, the activation of Wnt/β-catenin within the tumor cells has been demonstrated to result in the exclusion of T cells from the TME [518, 519]. Moreover, PTEN deficiency is linked to provoke PI3K-AKT pathway signaling and is connected to decreased presence of CD8^+^ T cells and unfavorable clinical outcomes from immunotherapy [520]. A recent study found that the loss of interferon regulatory factor 6 (Irf6) leads to resistance to immunotherapy, and its re-expression improves immunotherapy responses to PDAC [521]. All in all, the resistance to immunotherapy in pancreatic cancer is complex and influenced by both internal and external factors within the tumor. A sophisticated strategy of basic and translational/clinical research is needed to understand these mechanisms and identify tumor-specific resistance patterns.

### Immune-related adverse events (irAEs)

The irAEs are diverse and can affect any organ. Different immunotherapy regimens have unique toxicity patterns like CRS and neurologic toxicities, making understanding their mechanisms crucial [522–525]. However, a positive association exists between the occurrence of non-lethal irAEs and the response to ICB [526]. Mild (Grade 1–2) effects are observed in over 90% of patients, whereas severe (Grades 3–5) effects can occur in 20–60% of patients [527]. Studies highlight the role of certain immune cells such as CD8^+^ tissue-resident memory T cells and neutrophils, and cytokines like IFN-γ and IL-6 in causing immunotherapy-induced colitis [528–530]. Other mechanisms like loss of self-tolerance, molecular mimicry, and inflammation also contribute to irAEs. For example, in myocarditis, autoreactive T cells targeting specific peptides are activated, a process worsened by the release of self-antigens from dying tumor cells [531]. This is known as epitope spreading. Glucocorticoids are the main treatment for non-endocrine irAEs, and hormonal therapy is used for endocrine disorders. Intravenous immunoglobulins, plasma exchange, and monoclonal antibodies such as infliximab are employed for neurological, hematological, and persistent irAEs [22]. For ICB-induced colitis, fecal microbiota transplantation is utilized [532]. All in all, irAEs are life-threatening reactions that deserve special attention. Thus, it is crucial to formulate personalized strategies for patient categorization and potential biomarkers to investigate the dynamics and resolution timing of irAEs to identify.

### Scarcity of robust predictive biomarkers of response and toxicity

Individual biomarkers have been utilized to forecast responses to cancer immunotherapy. Both Microsatellite Instability-High (MSI-H) and Tumor Mutational Burden (TMB) have been associated with enhanced responses to ICB [533, 534]. Nonetheless, the efficacy of TMB as the only biomarker is restricted, as a low TMB can still elicit effective responses, and a high TMB does not assure a response to ICB [22]. Although immune-related biomarkers such as PD-L1, interferon signature, and TIL density have been found to have restrictions when used as the only biomarkers, it is important to note that even though there is an association between PD-L1 expression and improved outcomes in certain types of tumors, substantial responses can still be observed in tumors that do not express PD-L1 [535]. It has been proposed that CAFs, microbiomes, and exosomes derived from tumors could serve as potential biomarkers for tracking the response to immunotherapy in pancreatic cancer [536]. Furthermore, a study on pancreatic cancer patients who received PD-1 inhibitor-based therapies showed that a lower neutrophil-to-lymphocyte ratio predicted better tumor response [537]. Collectively, the progression of predictive biomarkers has been obstructed by the intricate interplay within the pancreatic tumor microenvironment. The field must comprehend these interactions and establish suitable assays for successful biomarker development and codifying combinatorial biomarker strategies. These approaches should be confirmed in upcoming clinical trials.

### Lack of integrated regulatory endpoints for cancer immunotherapy

Conventional methodologies for evaluating the efficacy of cancer immunotherapies, such as pembrolizumab and nivolumab, have demonstrated considerable utility [538]. These methodologies encompass metrics such as ORR, PFS, and OS, which have been instrumental in assessing therapeutic outcomes. Nevertheless, these conventional metrics exhibit limitations when applied to the evaluation of cancer immunotherapies. The primary objective of cancer immunotherapy is to induce a durable response and prolong survival, optimally quantified by examining the ‘tail’ of survival curves. However, the extant methodologies for this measurement are deficient [539]. In circumstances where an immunotherapy’s impact takes time to manifest and the rate of successful outcomes is not high, conventional benchmarks such as mPFS and median OS can provide deceptive early indications [473]. This becomes evident in the case of patients with MSI-H PDAC who underwent treatment with pembrolizumab. Despite a relatively low ORR of 18% and a mPFS of just 2.1 months, the responses proved to be quite durable, with a median response duration of 13.4 months [540]. All in all, continued collaboration and optimization of pancreatic cancer immunotherapy endpoints is needed to address the aforementioned issues.

### Lack of proper preclinical animal models

Preclinical models are crucial in cancer drug discovery for prioritizing targets and studying various aspects of treatment. These models have contributed to significant discoveries in cancer treatment and immunotherapy, including the effects of CTLA-4 and PD-L1/PD-1 blockade [539]. Nevertheless, the models commonly utilized do not always accurately represent the immune biology of human cancers [541]. This can be attributed to inter-tumoral and intra-tumoral heterogeneity, recapitulation of TME, and serial passaging of tumor cells [542]. For instance, the intricate interplay between tumor and stroma, along with the diverse traits of stromal elements, present substantial obstacles in accurately reproducing the pancreatic cancer microenvironment. Furthermore, the weak immunogenicity and the immunosuppressive characteristics of PDAC complicate preclinical modeling [542]. A problem with frequently utilized preclinical models is their dependence on the inoculation of cancer cell lines. The tumors that develop following this insertion often fail to accurately reproduce the immune context of the tumor, which plays a crucial role in shaping the immune response in human cancers [543]. Moreover, cancer in humans is thought to have evolved over the years, shaping its interaction with the immune response. Genetically engineered mouse models, developed by altering genes and inducing mutations, best represent this disease [544]. However, these models do not mimic the gradual mutation accumulation seen in human cancers, resulting in stable cancers that do not respond well to cancer immunotherapy [539]. Also, a significant obstacle in current attempts to comprehend the occurrence of irAEs is the absence of suitable preclinical animal models. There is a pressing need for the generation of animal models that accurately mimic irAEs, which would facilitate the detailed study of irAEs associated with pancreatic cancer immunotherapy [545]. Thus, there is an unmet need to further develop pancreatic cancer animal models with high-throughput techniques for better mimicking the human pancreas cancer features. It can pave the way for a rapid translation of preclinical findings into clinical settings.

## Conclusion and future directions

In summary, the paradigm shift brought about by immunotherapy is fundamentally altering our understanding of cancer treatment. This groundbreaking approach is now being implemented in clinical settings for a variety of solid cancers. Standard therapies have proven ineffective for patients with PDAC, but immunotherapy has demonstrated encouraging results in preclinical stages. Despite these promising results, immunotherapies still face fundamental challenges, which may limit their efficacy in clinical contexts. It is important to consider that each treatment modality has its advantages and disadvantages (Table 10). This article explored a wide range of immunotherapies, such as OVT, and adoptive cell transfer therapies including TCR-engineered T cells, CAR T-cell therapy, CAR NK cell therapy, and CIK cell therapy. Additionally, ICB, immunomodulators, cancer vaccines, and strategies targeting myeloid cells were discussed as potential avenues. Furthermore, this article provided the application of CRISPR/Cas9 technology and gut microbiome in pancreatic cancer immunotherapy. Lastly, strategies for enhancing the effectiveness of immunotherapy and the primary obstacles confronting pancreatic cancer immunotherapy were highlighted.

**Table 10:** Advantages and disadvantages/challenges of cancer immunotherapies

Treatment modality	Strategy	Advantages	Disadvantages/challenges	References
Oncolytic Virus Therapy		Inducing innate and tumor-specific adaptive immune responses Synergistic effects with whole host of other immunological agents (combination therapy) A versatile gene expression platform for the delivery of therapeutic genes	Poor efficiency in cold tumors Body’s anti-viral immune responses and neutralizing antibodies against oncolytic viruses Potential off-target infections Inadequate tumor cell tropism and transduction Lack of robust predictive biomarkers for patient selection to receive the treatment	[630–633]
Adoptive Cell Transfer Therapy	TIL therapy	Different T cell clones (polyclonal) to overcome tumor heterogeneity Better clinical efficacy in tumors with high mutation load A predominantly phenotype of effector memory T cell with cytotoxic functions expressing chemokine receptors for better homing at tumor tissues Low off-target toxicity	Tissue collection is cumbersome, expensive, and time-consuming Adverse events like thrombocytopenia, anemia, and febrile neutropenia High dose IL-2-induced toxicities. IL-2 is used to improve the in vivo survival and function of TILs Need to have sufficient quality and quantity of TILs for suitable therapeutic response	[634, 635]
	TCR therapy	Large repertoire of targetable tumor antigens Lower epitope density is required to induce T cell activation High avidity of TCR-T cells allows for eradicating several antigen-presenting tumor cells Allogeneic approaches and deleting both endogenous TCRα/β chains help the production of universal T cells to avoid the risk of GVHD	Choice of target antigen in terms of expression and immunogenicity Sensitization of TME for TCR therapy Unresponsiveness of T cells toward tumor antigen, reduced T cell fitness, short persistence of adoptively transferred T cells, and rapid exhaustion of engineered T cells On-target/Off-target toxicities and toxicity prediction Primary and secondary resistance to therapy Restriction to HLA alleles presenting the tumor epitope	[269, 581, 636–638]
	CAR T cell therapy	Rapid manufacturing, short treatment time, and usually a single infusion administration T cell activation in a HLA-unrestricted manner Promising outcomes in aggressive form of tumors like GBM and hematological malignancies Binding to the target with high affinity, T cell expansion, and generating memory T cells Available in several platforms like dual specific with many modifications and combinatorial therapies for better efficacy Existence of an acceptable range of antigenic targets	Antigen escape On-target off-tumor and increased risk of autoimmunity Limited CAR T cell trafficking and tumor infiltration Limited efficacy due to immunosuppressive TME CAR-T cell-associated toxicities like CRS and neurotoxicity Short-term persistence of CAR T cells in vivo GVHD incidence	[273, 582, 587, 639–641]
	CAR NK cell therapy	Robust response due to various activating receptors Low off-target response due to various inhibitory receptors Low risk of GVHD and CRS Allogeneic (haploidentical) NK cells can be used Provoking an ADCC-oriented cytotoxicity Off-the-shelf therapeutics and administration without HLA matching Recognizing the target in a HLA-unrestricted manner	Low rate of circulating NK cells NK cell expansion ex vivo is limited and tight Tight conditions in freezing and storage iPSC-derived NK cells often express low levels of endogenous CD16, leading to lower ADCC	[106, 642, 643]
	CIK cell therapy	HLA-unrestricted tumor-killing activity A heterogeneous population of CD3^+^ T lymphocytes that can kill heterogeneous tumor cells Applicable with many combinatorial treatment regimens Limited ability to induce GVHD There is no requirement for cellular selection during or after expansion in product manufacturing It has a remarkably safe profile	Limited understanding of pharmacological mechanisms Limited availability of CIK cell therapy in certain regions Heterogeneity of CIK cells makes it challenging to standardize CIK cell therapy	[327, 644]
ICT		Ability to conjugate with a variety of molecules like drugs and nanoparticles Available in several platforms like BsAbs, ScFvs, triabodies, and so on Combinatorial therapy with two or three different immune checkpoint inhibitors or other therapeutic agents Reinvigorating exhausted T cells/NK cell	Resistance to ICT Development of irAEs Failure of all patients to respond to the treatment The need for predictive biomarkers for optimal patient selection for gaining better clinical outcomes	[22, 517, 522–524, 527, 645–647]
Cancer Vaccine	Whole tumor cell vaccine	Containing a vast range of immunogenic epitopes Presenting all potential tumor antigens to immune system Providing a polyvalent anti-tumor immune responses No need for antigen selection during vaccine design	Poor immunogenicity No standardized dosing regimens	[648, 649]
	DC vaccine	The possibility of loading with a wide range of antigens like tumor lysate, peptides, RNA, DNA, neo-antigens, and viral antigens Promising safety, tolerability, and immunogenicity profile Producing a wide range of cytokines and chemokines by DCs Deploying a wide range of mechanisms by DCs, like cross-presentation, cross-dressing, antigen transfer, and MHCII-restricted presentation	Inefficient injected DCs migration and homing to lymph nodes Poor efficiency in immunosuppressive TME Optimization of in vitro culture and cytokine cocktails to achieve high-quality DCs Choosing the delivery route of the DC vaccine Selecting antigens and loading strategy for peptide-pulsed DCs in order to overcome tumor heterogeneity Need for effective combination therapies in advanced stages	[650–652]
	Peptide vaccine	Triggering a more focused immune response against immunodominant epitopes Containing both CD8^+^ T epitopes and CD4^+^ T cell epitopes Low risk of autoimmunity Direct presentation on MHC (short peptides)	Poor immunogenicity Peptide length-dependent vaccine efficacy Immunogenicity differences among different recombinant protein subunits Issues related to HLA restriction Need for appropriate adjuvants	[649, 652]
	Viral and bacterial vector vaccine	High immunogenicity and long-lasting immune responses Self adjuvanticity Long-lasting transgene expression	Potential for vector immunogenicity Specialized storage conditions Safety concerns related to oncogene activation in host’s cells and insertional mutagenesis	[652, 653]
	RNA vaccine	Inducing both humoral and cellular immune responses Providing systemic immunity against metastatic tumors Inducing long-term immunological memory Offering a personalized immunotherapy Lack of accidental infection and insertional mutagenesis Rapid translation of mRNA encoding TSA, TAAs, or personalized neo-antigens into protein in cytoplasm	Storage conditions and stability Issues related to design, production, and cost Lack of extensive preclinical and clinical trials Limited efficacy due to tumor heterogeneity and immunosuppressive TME Choosing the best vaccine administration route Need for biomarkers for monitoring the treatment response	[654, 655]
	DNA vaccine	Inducing both humoral and cellular immune responses Delivering multiple antigens simultaneously in the same construct at the same time Safety, stability, easy storage, and fast manufacturing/modifying on a large scale Providing native structure of protein	Limited migration into the cell nucleus due to extracellular and intracellular barriers Limited immunogenicity in clinical trials Integration of DNA into host genome Possibility for autoimmune reactions	[652, 656]
	Stem cell-based vaccine	Potential candidate to limit the chances of immune evasion due to shared epitopes between iPSCs and cancer cells Easy production at a short time Potential for triggering a pro-inflammatory profile in TME	Ethical constraints Limited studies and a need to further prove the results	[405]

ADCC: Antibody-dependent cellular cytotoxicity, BsAbs: Bispecific antibodies, CAR: Chimeric antigen receptor, CIK: Cytokine-induced killer, CRS: Cytokine release syndrome, DC: Dendritic cell, GBM: Glioblastoma, GVHD: Graft-versus-host disease, HLA: Human leukocyte antigen, ICT: Immune checkpoint therapy, iPSCs: Induced pluripotent stem cells, irAEs: Immune-related adverse events, MHC: Major histocompatibility complex, NK: Natural killer, ScFvs: Single-chain variable fragments, TAAs: T cell receptor, TCR: T cell receptor, TILs: Tumor-infiltrating lymphocytes, TME: Tumor microenvironment, TSAs: Tumor-specific antigens

The complex nature and heterogeneous composition of cellular elements in the pancreatic tumor microenvironment are of significant importance. There is a complex transition of cell populations as PDAC advances. Employing advanced methods such as single-cell sequencing and multi-omics analysis allows us to delve deeper into the immune cell profile in PDAC, pinpoint cells with higher precision, and chart the single-cell trajectories [546]. This advancement lays a stronger groundwork for developing immunotherapies that target the various elements of the TME.

As we progress in developing immunotherapeutic strategies for the treatment and management of PDAC, it is crucial to prioritize efforts that enhance patients' quality of life. Numerous trials employing immunotherapy in PDAC have had disappointing outcomes, primarily due to the immunosuppressive TME. Therefore, it is imperative to refine and improve existing immunotherapies to effectively address this significant challenge. Furthermore, it is essential to conduct further research on the efficacy of novel immunotherapy targets identified in preclinical studies, thereby validating their potential through human clinical trials. Overall, the open-ended research question remains unanswered as to why many patients with pancreatic cancer do not respond to immunotherapies.

The identification of novel and appropriate molecular targets for targeted immunotherapies is crucial for the success of this immunotherapy in treating pancreatic cancer. While CAR-based therapies have achieved impressive clinical responses in targeting cancer antigens, the efficacy of these therapies in solid cancers has been disappointing, in part due to antigen escape. Targeting heterogeneous pancreatic tumors with immunotherapies will require the identification of novel tumor-specific targets. Therefore, identifying novel and appropriate molecular targets for CAR T cell therapy is essential for the development of effective cancer treatments.

As our understanding of the complex interplay between the immune system and pancreatic cancer continues to evolve, the field of pancreatic cancer immunotherapy is positioned at the forefront of cutting-edge research. These groundbreaking domains of research, such as machine learning and artificial intelligence [547], mutant KRAS peptide-driven vaccines and personalized RNA neo-antigen vaccines [401, 548], single-cell multi-omics-oriented approaches [546, 549], and CRISPR/Cas-based RNA editing [550], are of utmost importance as they define the active research areas of the future, paving the way for gaining better clinical outcomes. Given the role of artificial intelligence in cancer research, researchers used machine learning to analyze complex tumor molecular data from pancreatic cancer patients and found that anti-CD40 therapy reduced T-cell exhaustion in the TME. They identified specific T-cell populations that correlated with improved DFS following anti-CD40 therapy, demonstrating the potential of machine learning in pancreatic cancer immunology research [547]. The creation of multiplexed effector guide arrays (MEGA) has made it possible to effectively control and regulate the T cell transcriptome through the use of CRISPR-Cas13d. With MEGA, genes can be suppressed in primary human T cells without any changes to the DNA, leading to improved T cell function and stronger anti-tumor capabilities. MEGA also enables the regulation of CAR activation and disruption of immunoregulatory metabolic pathways [550], providing a flexible and powerful tool for use in pancreatic cancer immunotherapy.

## Abbreviations

AEs: Adverse events
APC: Antigen-presenting cell
ATRA: All-trans retinoic acid
Breg: Regulatory B cell
BsAbs: Bispecific antibodies
CAR: Chimeric antigen receptor
CAF: Cancer-associated fibroblast
cDCs: Conventional dendritic cells
CRS: Cytokine release syndrome
CTL: Cytotoxic T lymphocyte
CTLA-4: Cytotoxic T-lymphocyte-associated protein 4
CSF1: Colony-stimulating factor 1
CSF-1R: Colony-stimulating factor 1 receptor
DC: Dendritic cell
DFS: Disease-free survival
ECM: Extracellular matrix
EGFR: Epidermal growth factor receptor
EMT: Epithelial-mesenchymal transition
FAPα: Fibroblast activation protein alpha
GM-CSF: Granulocyte–macrophage colony-stimulating factor
ICB: Immune checkpoint blockade
ICI: Immune checkpoint inhibitor
IDO: Indoleamine 2, 3-dioxygenase
IFN-I: Type I interferon
IFN-γ: Interferon-gamma
IL: Interleukin
iPSC: Induced pluripotent stem cell
LAG-3: Lymphocyte-activation gene 3
mAb: Monoclonal antibody
MDSC: Myeloid-derived suppressor cell
MHC: Major histocompatibility complex
MMP: Matrix metalloproteinase
mOS: Median overall survival
MSC: Mesenchymal stem cell
MSI-H: Microsatellite Instability-High
MSLN: Mesothelin
NK: Natural killer
ORR: Overall response rate
OS: Overall survival
OVT: Oncolytic virus therapy
PD-1: Programmed cell death protein 1
PDAC: Pancreatic ductal adenocarcinoma
PD-L1: Programmed death-ligand 1
PFS: Progression-free survival
PSC: Pancreatic stellate cell
STING: Stimulator of interferon genes
TA-MSCs: Tumor-associated mesenchymal stem cells
TAM: Tumor-associated macrophage
TCR: T-cell receptor
TGF-β: Transforming growth factor-beta
TH1: T helper 1
TH17: T helper 17
TH2: T helper 2
TIGIT: T cell immunoreceptor with Ig and ITIM domains
TIM-3: T-cell immunoglobulin and mucin domain 3
TIL: Tumor-infiltrating lymphocyte
TLR: Toll-like receptor
TMB: Tumor Mutational Burden
TME: Tumor microenvironment
Treg: Regulatory T cell
T-VEC: Talimogene laherparepvec
VEGF: Vascular endothelial growth factor
VISTA: V-domain Ig-containing suppressor of T-cell activation
